# Functional characterization of lysosomal interaction of Akt with VRK2

**DOI:** 10.1038/s41388-018-0330-0

**Published:** 2018-06-05

**Authors:** Noriyuki Hirata, Futoshi Suizu, Mami Matsuda-Lennikov, Tsutomu Tanaka, Tatsuma Edamura, Satoko Ishigaki, Thoria Donia, Pathrapol Lithanatudom, Chikashi Obuse, Toshihiko Iwanaga, Masayuki Noguchi

**Affiliations:** 1 Division of Cancer Biology, Institute for Genetic Medicine, Hokkaido University, Sapporo, Japan; 2 Chemistry Department, Faculty of Science, Tanta University, Tanta, Egypt; 3 Division of Molecular Life Science, Graduate School of Life Science, Hokkaido University, Sapporo, Japan; 4 Laboratory of Histology and Cytology, Hokkaido University Graduate School of Medicine, Sapporo, Japan; 5 Present Address: Department of Biology, Faculty of Science, Center of Excellence in Bioresources for Agriculture, Industry and Medicine, Chiang Mai University, Chiang Mai, 50200 Thailand

## Abstract

Serine–threonine kinase Akt (also known as PKB, protein kinase B), a core intracellular mediator of cell survival, is involved in various human cancers and has been suggested to play an important role in the regulation of autophagy in mammalian cells. Nonetheless, the physiological function of Akt in the lysosomes is currently unknown. We have reported previously that PtdIns(3)P-dependent lysosomal accumulation of the Akt–Phafin2 complex is a critical step for autophagy induction. Here, to characterize the molecular function of activated Akt in the lysosomes in the process of autophagy, we searched for the molecules that interact with the Akt complex at the lysosomes after induction of autophagy. By time-of-flight–mass spectrometry (TOF/MS) analysis, kinases of the VRK family, a unique serine–threonine family of kinases in the human kinome, were identified. VRK2 interacts with Akt1 and Akt2, but not with Akt3; the C terminus of Akt and the N terminus of VRK2 facilitate the interaction of Akt and VRK2 in mammalian cells. The kinase-dead form of VRK2A (KD VRK2A) failed to interact with Akt in coimmunoprecipitation assays. Bimolecular fluorescence complementation (BiFC) experiments showed that, in the lysosomes, Akt interacted with VRK2A but not with VRK2B or KD VRK2A. Immunofluorescent assays revealed that VRK2 and phosphorylated Akt accumulated in the lysosomes after autophagy induction. WT VRK2A, but not KD VRK2A or VRK2B, facilitated accumulation of phosphorylated Akt in the lysosomes. Downregulation of VRK2 abrogated the lysosomal accumulation of phosphorylated Akt and impaired nuclear localization of TFEB; these events coincided to inhibition of autophagy induction. The VRK2–Akt complex is required for control of lysosomal size, acidification, bacterial degradation, and for viral replication. Moreover, lysosomal VRK2–Akt controls cellular proliferation and mitochondrial outer-membrane stabilization. Given the roles of autophagy in the pathogenesis of human cancer, the current study provides a novel insight into the oncogenic activity of VRK2–Akt complexes in the lysosomes via modulation of autophagy.

## Introduction

Serine–threonine kinase Akt, a major downstream effector of the phosphatidylinositol-3 kinase (PI3K) pathway, regulates diverse cellular processes, including antiapoptotic processes, proliferation, the cell cycle, cytoskeletal organization, vesicle trafficking, and glucose transport [1–4]. Genetic and functional alterations of the Akt signaling pathways underlie the pathogenesis of a wide variety of human oncological diseases, glucose intolerance, viral infections, and autoimmune diseases [3–5]. A number of kinases, proto-oncogenes, and tumor-suppressor genes, including PI3K, PDK1, phosphatase and tensin homolog, Akt, TCL1, tuberous sclerosis complex 1/2 (TSC1/2), FOXO, mechanistic target of rapamycin (mTOR), or eukaryotic translation initiation factor 4E, are contained in this network [2, 3, 5].

Autophagy is an evolutionarily conserved mechanism in diverse life forms ranging from yeast to mammalian cells; it facilitates degradation and recycling of cellular components during cellular stress, such as nutrient starvation [6–8]. Although autophagy has been originally identified as a protective mechanism during starvation, it is also known as a mechanism controlling death of mammalian cells [9–13]. Therefore, autophagy is thought to underlie various processes in oncological diseases thereby modulating initiation and/or maintenance of cancers [14–20].

Lysosomes are intracellular membrane-bound organelles that orchestrate cellular catabolism and intracellular trafficking through autophagy [21–23]. A type of autophagy, so-called macroautophagy, sequesters cytosolic proteins or organelles within double-membrane vesicles forming autophagosomes, where protein molecules are degraded or recycled. In the process of autophagy, lysosomes and organelles involved in endocytic pathways fuse with autophagosomes, releasing their hydrolytic or proteolytic enzymes within autophagosomes and causing digestion or degradation of the engulfed macromolecules [22, 24–26].

The PI3K–Akt–mTOR pathway [3, 27, 28], which primarily mediates antiapoptotic signaling, has been suggested to play an important role in the regulation of macroautophagy, the most prevalent form of autophagy [29–33]. Recent studies further indicate that signaling molecules of the PI3K–Akt–mTOR pathway, including Akt, Vps34, mechanistic target of rapamycin complex 1 (mTORC1), mTORC2, glial fibrillary acidic protein, glycogen synthase kinase 3β (GSK3β), and TSC1/2, are present at the lysosomes [4, 34–39].

Three classes of PI3Ks (classes IA, IB, II, and III) are defined by their distinct substrate preferences [27]. Growth factor stimulation activates PI3K to produce PtdIns(3,4,5)P_3_, which recruits and activates Akt at the plasma membrane [40, 41]. Activation of Akt is believed to control autophagy at multiple steps [4, 19, 29, 34, 42]. Transcription factor EB (TFEB), a SITI homology and U-Box containing protein 1-controlled transcriptional regulator for autophagy [43], is also a target of phosphorylation by Akt at Ser467 in the control of autophagy induction independently of mTORC1 [44, 45]. Akt is known to phosphorylate and inhibit TSC1/2, leading to stabilization of Rheb GTPase, which in turn activates mTORC1, thus inhibiting autophagy [46]. Akt is also reported to directly phosphorylate ULK1 (ATG1) and Beclin 1 (ATG6), which control autophagy [4, 19, 46, 47].

We have demonstrated that Phafin2 interacts with Akt to facilitate its translocation to lysosomes, which control the induction of autophagy [36]. Subsequently, we found that the amounts of phosphorylated Akt remain high after Hank’s Balanced Salt Solution (HBSS) treatment intended to induce autophagy (see Fig. 5a, b). This observation prompted us to search for the molecules that interact with Akt in the lysosomes after the induction of autophagy. By means of time-of-flight–mass spectrometry (TOF/MS) analysis to search for Akt-interacting molecules in the lysosomes that are enriched after the induction of autophagy, kinases of the VRK family, a unique serine–threonine family of kinases in the human kinome, were identified (Supplement 1).

VRK harbors a catalytic domain homologous to that of the B1R kinase of the vaccinia virus; this domain acts at the early stage of viral infection to promote DNA replication [48–51]. The VRK family of kinases comprises three members: VRK1, VRK2, and VRK3 [52, 53] (Supplement 2a).

Both VRK1 and VRK2, but not VRK3, are catalytically active kinases. The physiological functions of VRK1 include cell cycle regulation through Myc, Fos, ATF2, CREB, mitotic histone H3, p53, and c-Jun [50, 54, 55]. The *VRK2* gene, located in chromosomal region 2p16-p15, is transcribed into two alternative splice variants in humans. VRK2A, the major form of VRK2, consists of 508 amino acid residues, with a transmembrane domain allowing it to localize to the endoplasmic reticulum and mitochondria [50, 55]. In contrast, VRK2B, the minor form, consists of 397 amino acid residues, lacks exon 13 of the transmembrane domain for lysosomal and/or endosomal localization, and is present in both the cytosol and the nucleus [55] (Supplement 2b).

Recent studies revealed that VRK2 mediates accumulation of polyglutamine aggregates by regulating the chaperon TCP-1 ring complex through GSK3β [56, 57]. Of note, VRK2A, but not VRK2B, contains a transmembrane domain for localization into membranes; however, no biological function for VRK2 at lysosomes has been described [50, 52].

Functional characterization of the VRK2–Akt interaction has clarified the functions of VRK2 in the maintenance of the sustained kinase activity of Akt in the lysosomes, which control not only various processes of autophagy but also cellular proliferation and survival. Context-dependent roles of autophagy in various characteristics of cancer pathogenesis have been suggested [13, 16, 20, 58]. Therefore, the present study provides a novel insight into the lysosomal function of serine–threonine kinase Akt, a core antiapoptotic regulator, which possibly mediates tumorigenesis by modulating autophagy.

## Results

### VRK2A interacts with Akt in the lysosomes

We have previously demonstrated that Phafin2 facilitates translocation of Akt to lysosomes, and this process is important for the induction of autophagy [36]. In this regard, we found that the amount of phosphorylated Akt (p-Akt) remains high after 4 h of HBSS treatment intended to induce autophagy, even though the cellular kinase activity of Akt is being inhibited (see Fig. 5a, b). To search for the Akt-interacting molecules that are enriched in the lysosomes after the induction of autophagy, Akt–Phafin2-transfected cells were treated with HBSS, which is widely used to induce autophagy in mammalian cells [59]. The lysosome fraction was then enriched as described elsewhere [36] and subjected to immunoprecipitation with an anti-FLAG antibody for TOF/MS analysis (Supplement 1a and b). Among the molecules that were enriched in the Akt immune complexes after the induction of autophagy, VRK2 was identified (Supplement 2a and b).

**Fig. 1:**
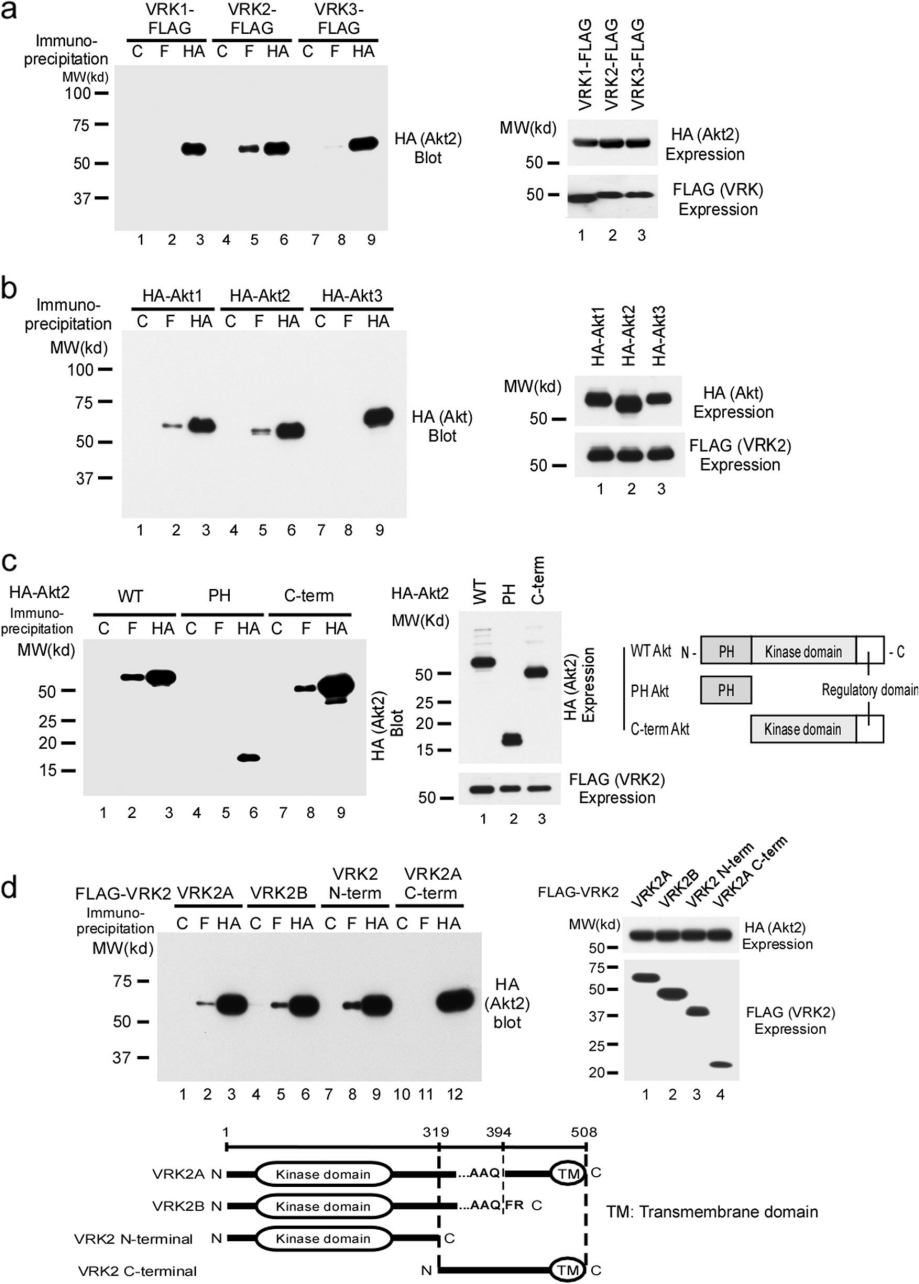
Interaction of VRK family of kinases with Akt in mammalian cells. **a** In coimmunoprecipitation experiments, FLAG-tagged VRK2 interacted with HA-tagged Akt in 293T cells. The expression of each construct is shown in the right-hand panel. **b** Among the three Akt isoforms, Akt1 and Akt2, but not Akt3, interacted with VRK2 in coimmunoprecipitation assays. The expression of each construct is shown in the right-hand panel. **c** The C terminus of Akt2 mediated the interaction with VRK2 in coimmunoprecipitation assays. The expression of each construct is shown in the right-hand panel. **d** WT VRK2A and WT VRK2B interacted with Akt through the N terminus of VRK2 in coimmunoprecipitation assays. A schematic representation of VRK2A and VRK2B is shown below. VRK2 has two alternatively spliced variants: VRK2A, containing 508 amino acid residues including the transmembrane domain, and VRK2B, containing 397 amino acid residues and lacking the transmembrane domain (Supplement 2a and b). Expression of each construct is shown in the right-hand panels

Among the three isoforms of VRK [53], VRK2, but not VRK1 or VRK3, interacted with Akt after coimmunoprecipitation (Fig. 1a). Both Akt1 and Akt2, but not Akt3, interacted with VRK2 (Fig. 1b). The Akt–VRK2 interaction was mediated by the C terminus of Akt (Fig. 1c) and the N terminus of VRK2 (Fig. 1d).

Two alternative splice variants of VRK2 (VRK2A and VRK2B) are known to exist in mammalian cells [49, 52] (Supplement 2a and b). Because VRK2 interacts with Akt through own N terminus, as expected, both VRK2A and VRK2B, which contain an identical N terminus, interacted with Akt2 (Fig. 1d).

**Fig. 2:**
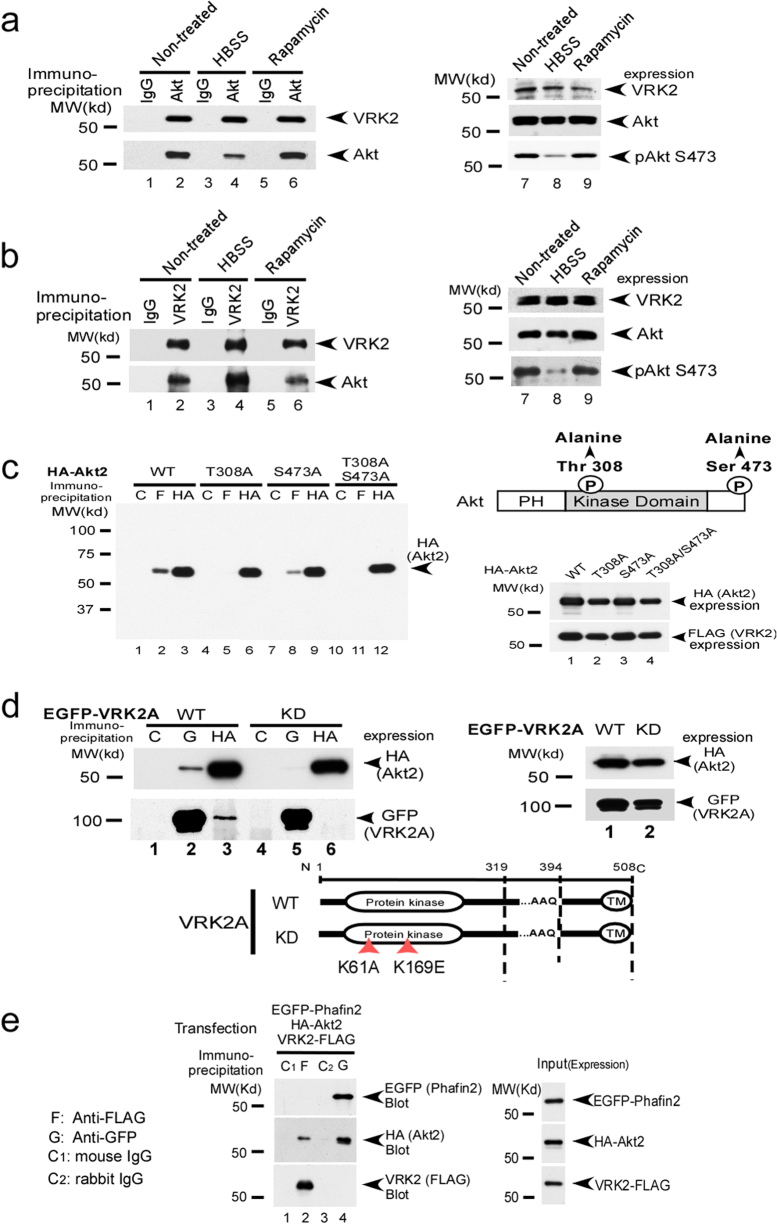
Interaction of Akt and VRK2 in mammalian cells. **a**, **b** HT-1080 cells (ATCC) were cultured in the presence of 25 μM chloroquine before rapamycin treatment (10 μM) or HBSS treatment for 4 h to induce autophagy. Endogenous Akt interacted with VRK2 in HT-1080 cells (lanes 2, 4, and 6 in the top panel: non-, HBSS-, or rapamycin-treated groups, respectively) in coimmunoprecipitation assays [76, 98, 99]. Expression of endogenous VRK2 under the three treatment conditions (top panel): total Akt (CST, #9272, second panel) is shown (right-hand panels). Note that the interaction of Akt with VRK2 appears to be enhanced after HBSS treatment, compared with the untreated cells (**a**, second panel, compare lane 2 with 4, and non- vs. HBSS-treated cells, respectively). **c** FLAG-VRK2, HA-Akt2, and EGFP-Phafin2 were cotransfected into 293T cells, and coimmunoprecipitation assays were performed [76]. Kinase-dead (KD) Akt (T308A or T308A/S473A) failed to interact with VRK2. Please note that S473A Akt only weakly interacts with VRK2, compared to T308A/S473A double mutant Akt. Expression and schematic structure of each construct are shown (right-hand panels). **d** KD VRK2 (K61A/K169E) interacted weakly with Akt as compared to the WT VRK2 in a coimmunoprecipitation assay using 293T cells. The schematic structure and expression of each construct are shown (right-hand panels). **e** FLAG-VRK2, HA-Akt2, and EGFP-Phafin2 were cotransfected into 293T cells, and coimmunoprecipitation assays were performed with anti-FLAG and anti-GFP antibodies and immunoblotting with an anti-GFP antibody (top panel), anti-HA antibody (second panel), and anti-FLAG antibody (third panel). Akt interacted with Phafin2 and VRK2 (lanes 2 and 4, middle panel), but no interaction between Phafin2 and VRK2 was detected (third panel)

Furthermore, endogenous Akt interacted with VRK2 in HT-1080 cells (Fig. 2a). The interaction of Akt with VRK2 was confirmed when the samples were processed by immunoprecipitation with an anti-VRK2 antibody and immunoblotted with an anti-Akt antibody (Fig. 2b).

We next tested whether phosphorylation of Akt could affect its interaction with VRK2. VRK2 preferentially interacted with wild-type (WT) Akt, only weakly with the S473A mutant, and failed to interact with the T308A or T308A/S473A Akt mutant (Fig. 2c). In contrast, WT VRK2A, but not kinase-dead (KD) VRK2A (K61A/K169E), interacted with Akt well (Fig. 2d).

Because VRK2 was identified by TOF/MS analysis in the cells transfected with Akt2 and Phafin2 [36], we next determined whether VRK2 interacts with Akt directly or possibly through Phafin2 in coimmunoprecipitation assays. In 293T cells transfected with Akt, VRK2, and Phafin2, we observed Akt interaction with Phafin2 and VRK2, but no interaction between Phafin2 and VRK2 was detected (Fig. 2e). This observation suggested that VRK2 directly interacted with Akt, but not through Phafin2.

### Kinase activities of both VRK2A and Akt are important for lysosomal localization

**Fig. 3:**
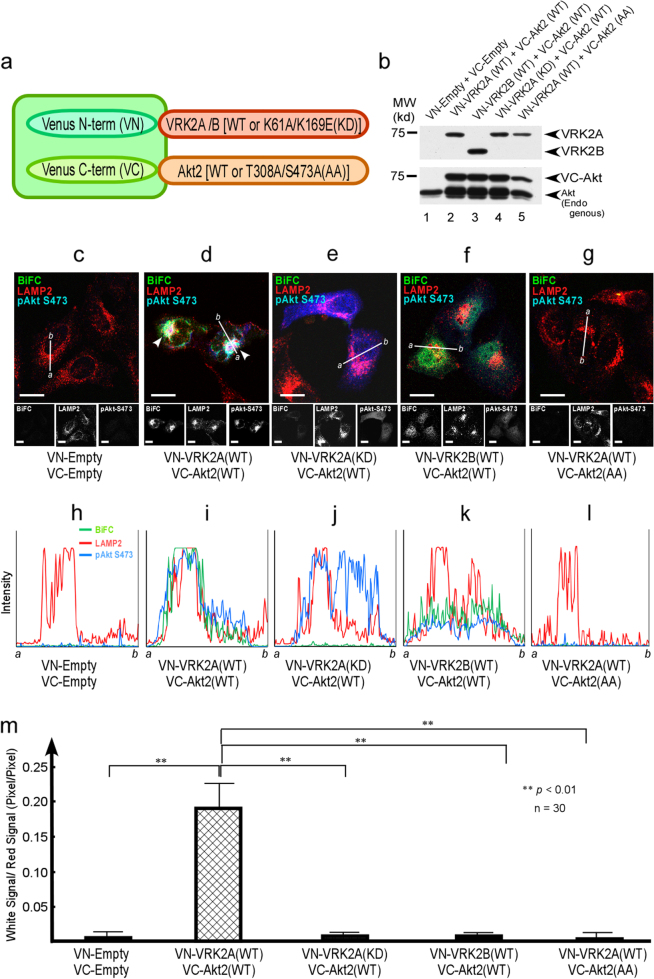
Kinase activities of both Akt and VRK2 are important for lysosomal accumulation of VRK2-Akt complexes. **a** By BiFC assays [36, 101], intracellular spatial localization of Akt and VRK2A with lysosomes was examined. Schematic view of BiFC is shown. **b** Equal levels of expression of the constructs used in the study were confirmed by western blotting. **c**–**g** HeLa cells were transfected with pCMV-VN-Empty, pCMV-VC-Empty, pCMV-VN-VRK2A, pCMV-VC-Akt2, pCMV-VN-VRK2A-Kinase Dead, or pCMV-VC-Akt2-T308A/S473A as indicated. WT Akt–WT VRK2A complexes (**d**, green), but not WT Akt–VRK2B complexes (**f**), were localized to the lysosomes, as determined by LAMP2 staining (red) with Akt phosphorylated at Ser473 (**d**, arrowheads). Formation of VRK2A–Akt complexes was abrogated when KD VRK2A (K61A/K169E, **e**) or KD Akt (T308A/S473A, **g**) or VRK2B (**f**) that lacks the lysosomal localization signal were introduced. Note that VRK2B, which lacks the lysosomal localization signal, showed weak BiFC (**f**, green color) but failed to colocalize with p-Akt (**f**, blue) in the lysosomes. **h**–**l** To determine the relative intensities of these signals, fluorescence intensities of BiFC (green) and Alexa Fluor 568 (LAMP2, red), and p-Ser473 Akt (Blue) along the line (*a*–*b*) were plotted [36]. Green signals (BiFC, interaction of WT VRK2A and WT Akt) and red signals (LAMP2, lysosomes) overlapped with the blue signals (p-Ser Akt) only in the cells transfected with the combination of WT VRK2A and WT Akt (**d**, **i**). The white scale bar represents 10 μm. **m** For the quantification of BiFC data, [white signals (combination of BiFC with p-Ser473 Akt)] and LAMP2 (red signals) from 30 cells are shown as a bar graph. White signals, which represent areas of colocalization of BiFC (WT VRK2 and WT Akt, green signals) and p-Ser473 Akt (blue signals), are predominantly present (as white signals after merging) in the cells transfected with the combination of WT VRK2A and WT Akt in the lysosomes (second bar from the left, also see **d**, **i**)

We next tested whether phosphorylation is required for formation of the Akt–VRK2 complex in the lysosomes. All the VRK2-BiFC and Akt-BiFC constructs were equally expressed in these cells, as shown by immunoblotting (Fig. 3a, b). BiFC analysis revealed that only WT Akt (Fig. 3d), but not KD VRK2A (K61A/K169E, Fig. 3e) or KD Akt (T308A/S473A, Fig. 3g), formed complexes that colocalized with positive staining for p-Ser473 Akt (Fig. 3d) without a detectable background (Fig. 3c). VRK2B, which lacks the lysosomal localization signal, showed weak BiFC signals (Fig. 3f, green) but failed to colocalize with p-Akt in the lysosomes (Fig. 3f, blue).

Colocalization of BiFC (WT VRK2 and WT Akt, green signal) and p-Ser473 Akt (blue signal) was seen (as a white signal) only in the cells transfected with the combination of WT VRK2A and WT Akt in the lysosomes (LAMP2, red signal), with the relative intensities of these signals (Fig. 3h–l) and quantification presented as a bar graph (Fig. 3m). These results suggested that the kinase activities of both VRK2A and Akt are important not only for formation of the Akt–VRK2 complex but also for lysosomal accumulation of activated Akt.

### Local kinase activity of Akt in the lysosomes is crucial for the induction of autophagy

**Fig. 4:**
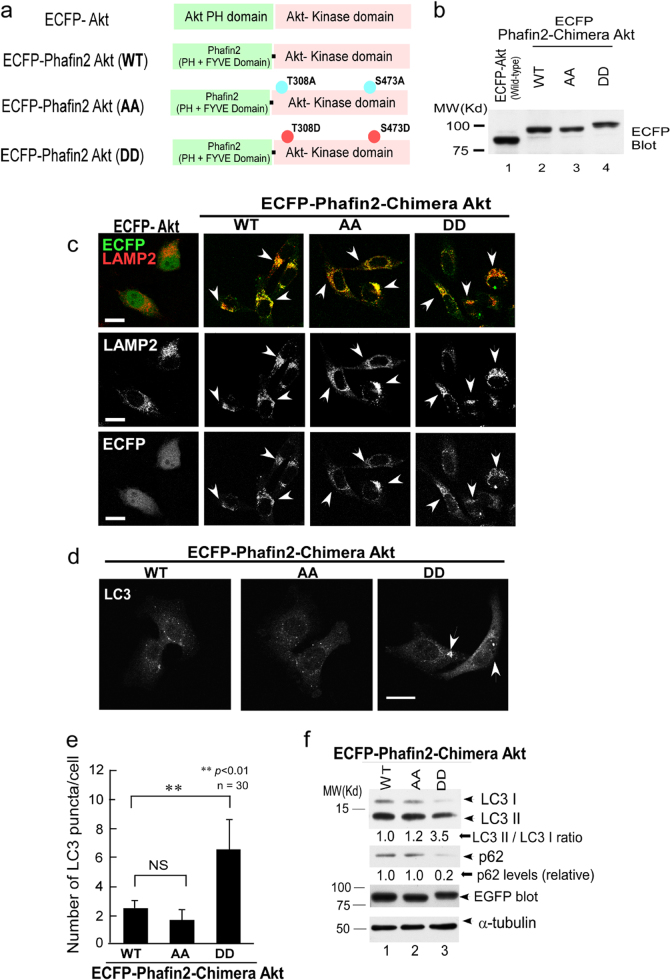
Local kinase activity of Akt in the lysosomes is crucial for induction of autophagy. **a** ECFP-Akt2 and ECFP-Phafin2-fused Akt constructs (WT, AA, and DD) used in this study are shown schematically. **b** Expression of Phafin2-fused Akt constructs is confirmed by western blotting. **c** Constructs of ECFP-Phafin2 fused to the C terminus of Akt (WT, AA, or DD version: second, third, and fourth panels, respectively), but not ECFP-Akt (Phafin2-free construct, left-hand panels), efficiently localized to lysosomes as determined by colocalization with LAMP2. **d** After HBSS treatment to induce autophagy, a constitutively active form of Phafin2-fused Akt (DD, right-hand panels) yielded more numerous LC3 puncta as compared to the AA version (left-hand panels). Please note that untreated cells showed no obvious differences in the LC3 staining. **e** Quantification of LC3 puncta normalized to white signals (bottom panels from **c**) is shown (*n* = 30) with statistics. **f** After HBSS treatment to induce autophagy, a constitutively active form of Phafin2-fused Akt (DD, lanes 3, before and after the induction of autophagy, respectively) resulted in greater LC3II/I conversion as compared to KD Akt (AA, lanes 2, before and after the induction of autophagy, respectively), which is associated with diminished levels of p62 (second panels)

Akt is suggested to control autophagy at multiple steps [4, 19, 29, 34, 42]. Therefore, we tested whether greater accumulation of activated Akt in the lysosomes may affect the induction of autophagy. Previously, we have shown that Phafin2 is translocated to lysosomes in a PtdIns(3)P-dependent manner [36, 60]. Phafin2 contains two PtdIns(3)P-binding motifs: the N-terminal PH (pleckstrin homology) domain and C-terminal FYVE (Fab1, YOTB, Vac1, and EEA1) domain for lysosomal localization [61, 62]. To examine the specific roles of the local kinase activity of Akt in the lysosomes for the induction of autophagy, we fused Phafin2 to the Akt C terminus [(WT, double alanine mutant (AA-Akt), or double aspartic acid mutant (DD-Akt)] to enforce the lysosomal localization (Fig. 4a, b). As expected, Phafin2-fused chimeric Akt2-CT, but not WT Akt, localized to lysosomes (Fig. 4c). After HBSS treatment, DD-Akt mutant–transfected cells showed more LC3 puncta as compared to AA-Akt mutant–transfected cells (Fig. 4d, e). Furthermore, autophagy could be induced only in DD-Akt–transfected cells, but not in AA-Akt–transfected cells, as revealed by LC3II conversion and by diminished levels of p62 (Fig. 4f). This observation supported the possible functions of the local kinase activity of Akt in the lysosomes for control of the induction of autophagy.

### Endogenous VRK2 and Akt accumulate in the lysosomes after induction of autophagy

**Fig. 5:**
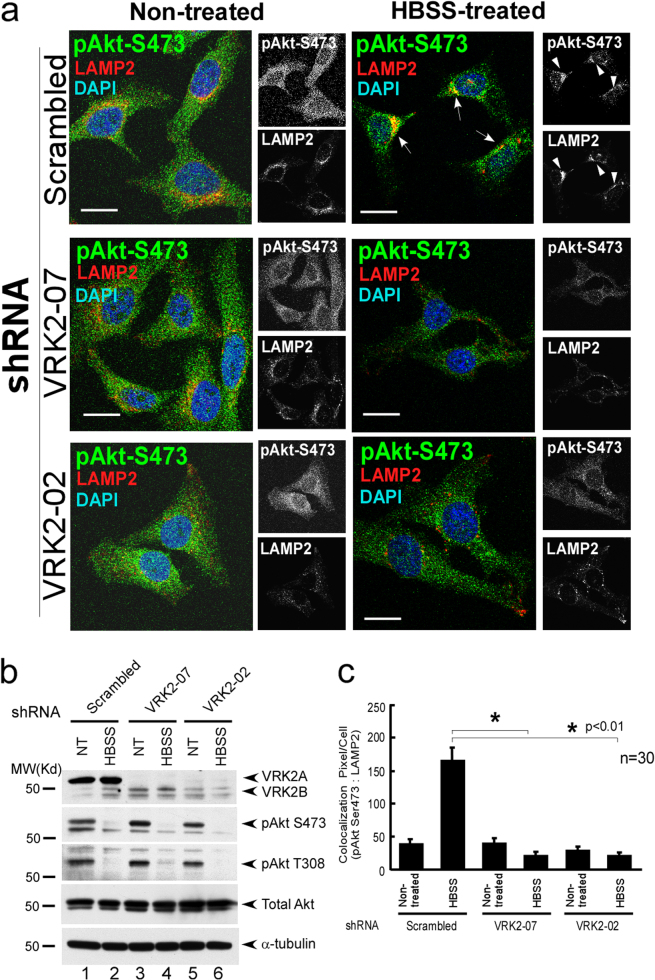
Inhibition of VRK2 expression attenuates accumulation of p-Akt in the lysosomes. **a** HeLa cells were fixed with formaldehyde, stained with the indicated antibodies and DAPI, and examined by confocal microscopy [98, 99]. After the induction of autophagy by HBSS, p-Akt [p-Ser473 Akt, CST 4060 (D9E) antibody] was costained with a lysosome marker (LAMP2) in the perinuclear region after HBSS treatment (first panels from the top, arrowheads). Notably, inhibition of VRK2 expression by two shRNAs (shRNA-07 or shRNA-02), which drastically inhibited the expression of endogenous VRK2 (see Fig. 4b, top panel, lanes 1, 3, and 5), abrogated the lysosomal accumulation of Akt phosphorylated at Ser473 (second and third panels from the top). **b** Lentiviral supernatants that contain the shRNAs specific for VRK2 were collected as described previously [102–104]. The stable cell lines that expressed shRNAs were selected in the presence of puromycin. shRNA targeting VRK2 (shRNA-07 or shRNA-02) attenuated the expression of endogenous VRK2 before or after the induction of autophagy by HBSS treatment in HeLa cells (top panel). Notably, general cellular levels of Akt phosphorylation (p-Ser473 and p-Thr308) remained high after the inhibition of VRK2 expression by shRNAs (lanes 3 and 5, third and second panels from the top, p-Thr308 and p-Ser473, respectively). **c** Quantification of colocalized signals from panels and analysis by Student’s *t*-test

Having demonstrated that ectopic Akt interacts with VRK2 (Figs. 1 and 2), we next examined whether endogenous VRK2 and Akt could colocalize to lysosomes after the induction of autophagy. Immunofluorescent assays showed that phosphorylated Akt accumulated in the lysosomes as determined by costaining for LAMP2 after 8h HBSS treatment in HeLa cells (Fig. 5a). Inhibition of VRK2 by short hairpin RNAs (shRNAs) attenuated the lysosomal accumulation of p-Akt (Fig. 5a, c for quantification).

Notably, in VRK2 knockdown cells, cellular levels of both total Akt and p-Akt remained constant in the absence of VRK2 before HBSS treatment (Fig. 5b). After HBSS treatment to induce autophagy, cellular levels of Akt kinase activity decreased in both control and VRK2 knockdown cells (Fig. 5b) without affecting the levels of mTOR or p-mTOR, as shown by immunostaining of the cells and by immunoblotting (Supplement 3a and b). Furthermore, increasing the amount of VRK2 via transfection did not alter either p-mTOR or total mTOR amounts according to immunoblots (Supplement 3c).

Endogenous VRK2 accumulated at the lysosomes after the induction of autophagy as determined by the coimmunostaining for LAMP2 and VRK2 (Supplement 3a, middle panels). Moreover, endogenous VRK2 and p-Akt colocalized after the induction of autophagy (Supplement 4, lower panels). VRK2 was enriched in the lysosomes after the induction of autophagy in the cells transfected with both Akt and Phafin2 (Supplement 1a). Inhibition of Phafin2 expression by small interfering RNA (siRNA) abrogated the lysosomal accumulation of VRK2, indicating that Phafin2 is required for the accumulation of VRK2 at the lysosomes (Supplement 5a and 5b for quantification). Taken together, these results showed that VRK2 is important for the sustained levels of the activated Akt kinase at lysosomes after HBSS treatment and the induction of autophagy.

### The lysosomal VRK2–Akt complex controls the induction of autophagy

**Fig. 6:**
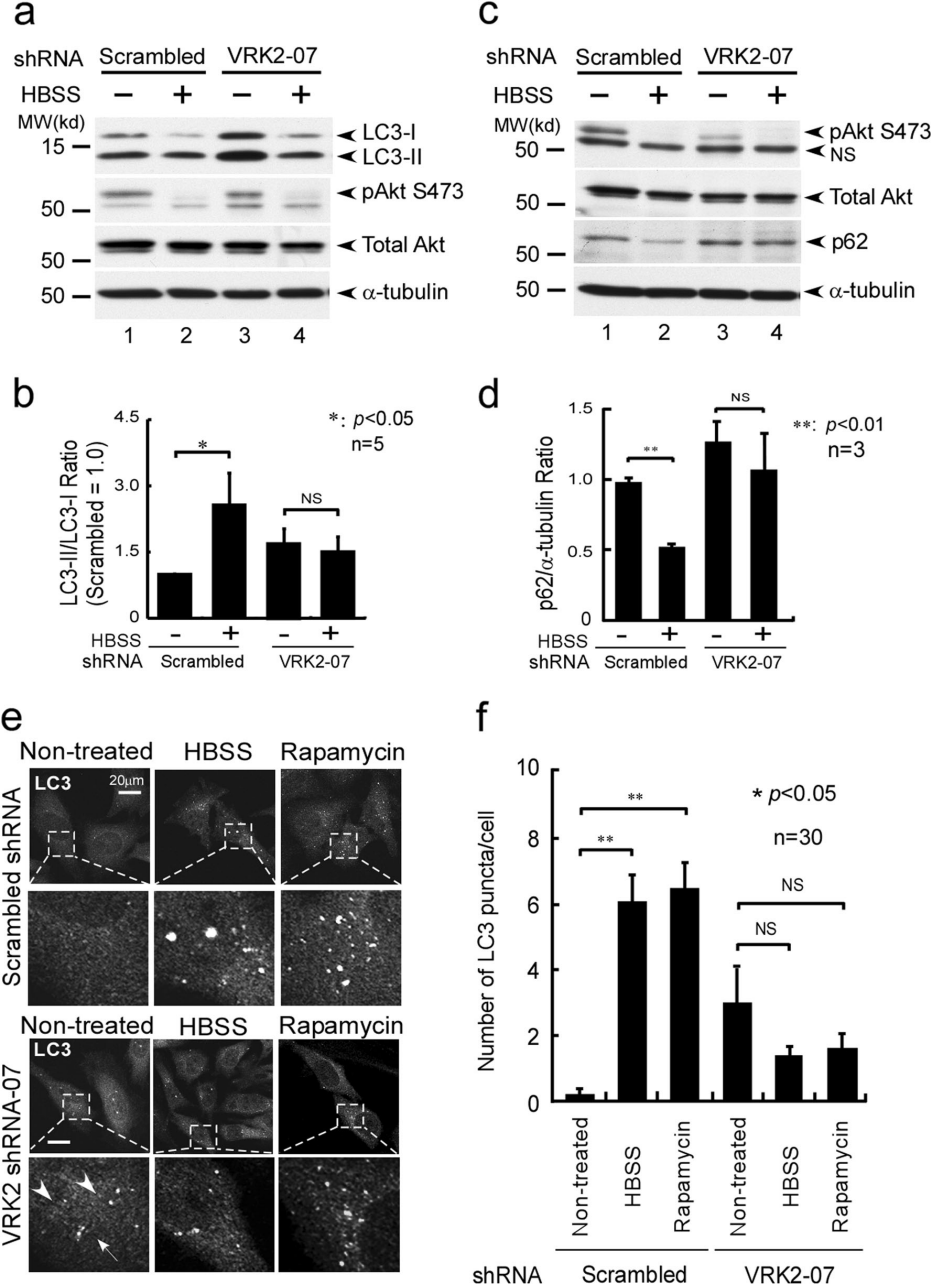
VRK2 is important for the induction of autophagy. **a** HeLa cells (ATCC) transduced with VRK2 shRNA or control (scrambled) shRNA were washed twice with HBSS, then incubated with HBSS for additional 8 h to induce autophagy. Downregulation of VRK2 by shRNAs increased the amounts of lipidated forms of LC3 (LC3II) according to the ratio of LC3II/LC3I conversion (top panel, compare lanes 1 and 3). Notably, however, further induction of autophagy was inhibited as revealed by the compromised LC3II/LC3I conversion after HBSS-induced autophagy (top panel, lane 4). **b** Quantification of LC3II/LC3I conversion (**a**) from five independent VRK2 shRNA experiments is shown. **c** Inhibition of VRK2 by shRNA abrogated the induction of autophagy as assessed by the levels of p62 (third panel from the top). **d** Quantification of the relative expression of p62 (**c**) in three independent experiments is shown with statistical analysis by Student’s *t*-test. **e** Inhibition of VRK2 expression by VRK2 shRNAs, which caused a modest increase in LC3 puncta numbers in untreated cells (left-hand panels, the fourth panel from the top, arrowheads) or attenuated the induction of autophagy by HBSS (middle panels) or rapamycin-treated cells (right-hand panels). **f** Quantification of the numbers of LC3 puncta per cell from 30 samples is shown with statistical analysis by Student’s *t*-test

The observation that VRK2 participates in the maintenance of accumulation of p-Akt in the lysosomes prompted us to test whether VRK2 affects the induction of autophagy. Inhibition of VRK2 modestly promoted the induction of autophagy (Fig. 6a). Notably, however, further induction of autophagy by HBSS treatment could be abrogated as assessed by the LC3II conversion [59] (Fig. 6a, b). These observations are consistent with the finding that VRK2 shRNA attenuates the inhibition of degradation of p62 [59, 63, 64] (Fig. 6c, d). Similarly, inhibition of VRK2 resulted in modest enhancement of LC3 puncta formation; however, neither HBSS nor rapamycin treatment further enhanced the induction of autophagy (Fig. 6e, f). Altogether, these observations suggested that functional interaction of VRK2 with Akt participates in the control over autophagy.

### VRK2–Akt controls lysosomal size and acidification and the hydrolytic activity of cathepsin D

**Fig. 7:**
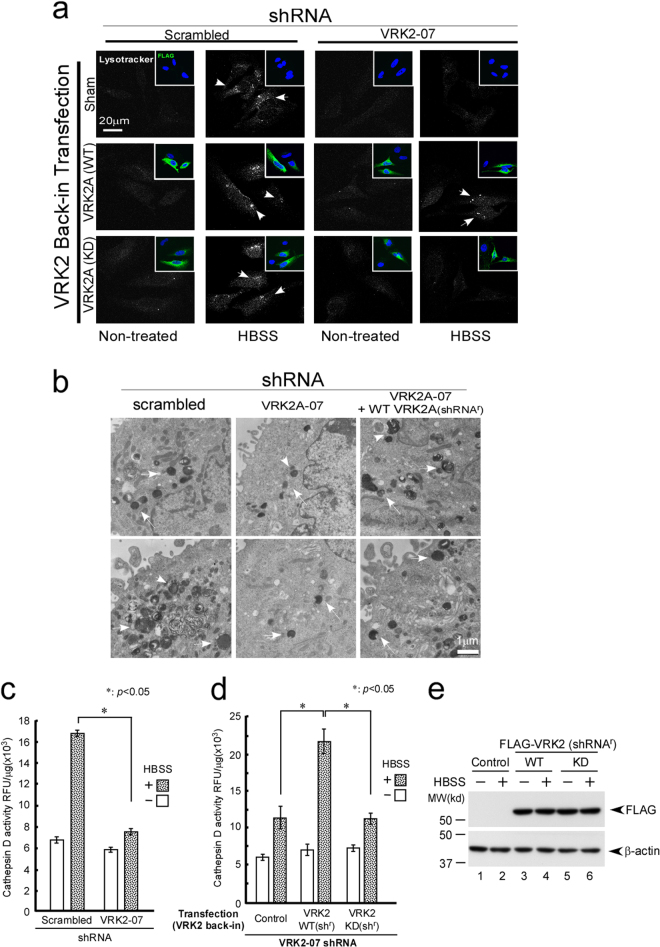
VRK2 controls lysosomal acidification, size, and hydrolytic activity of cathepsin D. **a** HeLa cells (ATCC) with or without shRNA transfection for a VRK2 knockdown (scrambled, shRNA-07) were incubated with HBSS for 8 h or not treated (DMSO). The cells were stained with LysoTracker Red DND-99 (Thermo Fisher) and an anti-FLAG antibody (green) for confocal microscopy [98]. Lysosomal acidification, as assessed by LysoTracker (white arrowheads), was attenuated by VRK2 shRNA (top panel, far right). For the reconstruction experiments shown in Fig. 6, the indicated shRNA-resistant constructs were transfected by means of Lipofectamine 3000 (Invitrogen). Acidification of lysosomes could be restored only in the cells in which WT VRK2A (VRK2A-WT, arrowheads in middle panels, far right) is expressed as shown by FLAG staining but not in KD VRK2A-expressing cells (bottom panels, far right) as determined by FLAG immunostaining shown in the inset (green cells). **b** HeLa cells (ATCC) transfected with VRK2 shRNA were treated with HBSS for 8 h to induce autophagy for TEM as described elsewhere [36]. The expression of VRK2A was confirmed by immunoblotting (see Fig. 4a). Decreased size and laminar structure of the lysosomes (arrowheads) were observed by TEM in cells transfected with VRK2A shRNA (middle panels), as compared to the control shRNA (left panels). The normal properties could be partially restored by reintroduction of VRK2 shRNA-resistant VRK2A (right-hand panels). The scale bar represents 1 μm. **c** Cathepsin D activity was suppressed by the elimination of VRK2 in VRK2 shRNA-transfected cells. HeLa cells with a stable VRK2 knockdown (VRK2 shRNA 07) were transfected with shRNA-resistant WT or KD VRK2. After HBSS treatment for 8 h, cells were harvested and lysed, and cathepsin D assays were performed using the SensoLyte^TM^ Cathepsin D Assay Kit (ANASPEC, Inc., CA, USA). The data shown are normalized to protein concentration measured by a Bio-Rad Protein Assay (relative fluorescence units [RFU]/[μg protein]). **d** For the reconstruction experiments, the indicated shRNA-resistant constructs were transfected by means of Lipofectamine 3000 (Invitrogen). Reintroduction of shRNA-resistant WT, but not KD VRK2A, restored cathepsin D activity. **e** Expression levels of VRK2A (WT and KD) were confirmed by western blotting

Recent studies indicate that the Akt–mTORC1 pathway is involved in the regulation of lysosomal acidification [25, 65, 66]. The observation that lysosomal interaction of the VRK2–Akt complex controls autophagy prompted us to investigate the involvement of VRK2 in the regulation of lysosomal acidification and subsequent hydrolytic activity of the enzymes. As predicted, impaired lysosomal acidification was observed (using LysoTracker) after HBSS (Fig. 7a) or rapamycin treatment (Supplement 7a) of VRK2 shRNA–transduced cells; this impairment coincided with inhibition of conversion of LC3I to LC3II (Fig. 6a). Notably, reintroduction of WT VRK2A, but not KD VRK2A, restored lysosomal acidification after HBSS treatment (Fig. 7a), thereby supporting the importance of VRK2 kinase activity for lysosomal acidification. Transmission electron microscopy (TEM) showed a decrease in the size of the lysosomes and diminished laminar structure in HeLa cells stably transduced with VRK2A shRNA as compared with control cells (Fig. 7b). Recently, TFEB was identified as a core transcriptional regulator of autophagy induction [43, 67]. TFEB did not accumulate in the nucleus in the VRK2 knockdown cells after HBSS treatment as compared to control cells (Supplement 6a and b). The observation of a mobility shift in a phos-tag sodium dodecyl sulfate polyacrylamide gel electrophoresis assay is due to phosphorylation of TFEB by Akt or possibly by mTORC (Supplement 6c) [44, 45, 68, 69]. Taken together, these observations supported the idea that impaired biogenesis of lysosomes could be in part due to impaired dephosphorylation of TFEB. The decreased size of lysosomes could be partially restored by shRNA-resistant VRK2A^r^, and this finding supported the necessity of VRK2 for this process.

Lysosomal acidification is known to be important for the hydrolytic activity of lysosomal enzymes. Lysosomes contain cathepsin D, a proteolytic enzyme that is activated by acidification of lysosomes [25]. Indeed, cathepsin D activity was suppressed by downregulation of VRK2 in VRK2 shRNA–transduced cells (Fig. 7c). Moreover, shRNA-resistant WT VRK2A, but not KD VRK2A, restored cathepsin D activity after HBSS treatment, suggesting that kinase activity of VRK2 is required for the hydrolytic activity (Fig. 7d, e). It is worth noting that KD VRK2A, which failed to interact with Akt in coimmunoprecipitation assays (Fig. 2d) and in BiFC experiments (Fig. 3g), also manifested itself as an Akt interaction–defective mutant in the functional experiment. These results indicated that the kinase activity of VRK2 plays a role in the control over lysosomal acidification and subsequent hydrolytic activity.

### The VRK2–Akt complex controls digestion of bacteria and viral replication

**Fig. 8:**
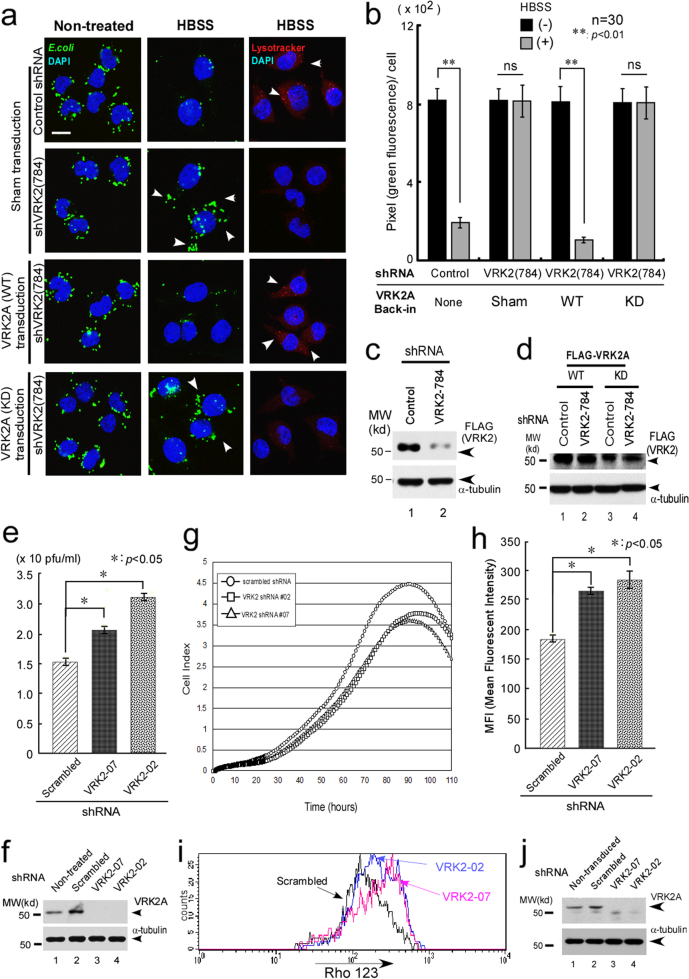
VRK2 is important for the induction of autophagy, elimination of intracellular bacteria, and for viral replication. **a** J774.1 cells (ATCC) stably transduced with lentiviral VRK2 shRNA (VRK2-784 for mouse VRK2 or EGFP as a control shRNA) were generated as described elsewhere [36]. Among J774.1 cell lines, the initial uptake of fluorescent bacteria (green signals) appeared to be similar in the VRK2 shRNA–transduced cells (784, second row from the top on the left-hand panels) compared to control shRNA-transduced cells (top panels, left side). After 4 h of HBSS treatment to induce autophagy, elimination of bacteria was impaired (according to decreased green signals) in VRK2 shRNA-transduced cells (middle panels, arrowheads); these phenomena coincided with the impairment of lysosomal acidification (LysoTracker, red signals in right-hand panels). Reintroduction of WT VRK2 (third panels from the top), but not KD VRK2 (fourth panels from the top), restored the elimination of bacteria (decreased green signals, third panels from the top, middle row) and acidification (increased red signals, third panels from the top, right-hand panels, arrowheads). The scale bar represents 10 μm. **b** Quantification of fluorescent bacteria with statistical analysis by Student’s *t*-test. **c**, **d** Expression levels of VRK2 protein in J774.1 cells were verified by immunoblotting with EGFP shRNA as a control. **e** In A549 cells transduced with VRK2 shRNA, viral replication was assessed by plaque assays as described elsewhere [105]. Inhibition of VRK2 expression resulted in the enhancement of the replication of PR8 influenza virus according to statistical analysis. **f** Downregulation of VRK2 proteins was verified by immunoblotting. **g** Proliferation assays were analyzed by means of an xCelligence system (Real Time Cell Analyzer, Roche) using stably VRK2 shRNA (designed as #02 or #07) expressing 293T cells [98]. The knockdown of VRK2 by shRNA (#02 and #07) suppressed natural cell growth. Results were consistent among HeLa cell clones expressing VRK2 shRNAs. **h** Inhibition of VRK2A expression by VRK2 shRNA (#02 or #07) significantly increased mitochondrial depolarization assessed by Rhodamine 123 staining. Results were consistent in two independent experiments. **i** A representative histogram of Rhodamine 123 profiles after transfection with VRK2 shRNA (#02 and #07 with scrambled shRNA as a control). **j** Downregulation of the VRK2 protein by shRNA in 293T cells (**g**–**i**) was verified by immunoblotting

Lysosomes are known to degrade phagocytosed infectious particles through endocytic and autophagic pathways [21]. Inhibition of VRK2 in J774.1 cells (Fig. 8c) did not affect the initial uptake of fluorescent *Escherichia coli* (Fig. 8a). After HBSS treatment, the cells transfected with VRK2 shRNA failed to eliminate the fluorescent *E. coli*. This result coincided with the impairment of lysosomal acidification (Fig. 8a, red signal arrows). In addition, shRNA-resistant WT VRK2, but not KD VRK2, restored the elimination of bacteria and acidification, suggesting that the VRK2 kinase activity is required for these processes (Fig. 8a, b).

In the course of viral infection, viruses internalized by endocytosis are delivered to endosomes, where inhibition of endosomal and lysosomal degradation has been shown to increase their infectivity [70, 71]. We chose PR8 as a model system for studying the influenza virus infection. Inhibition of the expression of VRK2 by shRNA as determined by western blotting (Fig. 8f) significantly enhanced replication of PR8 influenza virus in plaque assays involving A549 cells (Fig. 8e). These results showed that lysosomal VRK2–Akt interaction performs a critical function in host cell defense in the course of infection with the influenza virus.

### VRK2–Akt is important for cellular proliferation and survival

Physiological roles of VRK2 in cellular proliferation and survival have been suggested [50, 55]. Likewise, we showed that the knockdown of VRK2 leads to modest suppression of cellular proliferation (Fig. 8g). Conversely, overexpression of VRK2A enhanced natural cell growth (Supplement 7b).

VRK2 is known to regulate apoptosis by interaction with Bcl-xL or Epstein–Barr virus BHRF1, a homolog of Bcl-2 for cell survival [72, 73]. Mitochondrial transmembrane potential (MTP) is crucial for regulation of the cell death machinery [74], and Akt regulates this process [75, 76]. Furthermore, autophagic and apoptotic pathways are suggested to converge on mitochondria [13].

According to these reports, we next examined the possible roles of VRK2 in the cell survival machinery. Downregulation of VRK2 significantly increased mitochondrial potential as shown by Rhodamine 123 staining as a consequence of destabilization of MTP (Fig. 8h, i). Inhibition of proliferative responses by the knockdown of VRK2 is consistent with another report, indicating that higher levels of VRK2 expression are present in a subgroup of primary high-grade astrocytoma cells [77]. Autophagy is believed to participate in initiation and maintenance of cancer [15–20]. Therefore, this observation pointed to a new property (oncogenic function) of the VRK2–Akt complex in the lysosomes.

## Discussion

Recent studies revealed that major signaling molecules of the PI3K–Akt cascade, including mTORC1, Akt, mTORC2, GSK3β, and TSC, are present at the lysosomes, the major organelles that execute autophagy [4, 34–39]. Nonetheless, the exact roles of Akt in the lysosomes are not well characterized [4, 13, 32, 42]. In the present study, VRK2 was identified among the molecules that associate with Akt at the lysosomes after the induction of autophagy. The functions of Akt in the regulation of autophagy have not been fully clarified, and Akt is known to phosphorylate and inhibit TSC1/2, thus leading to stabilization of Rheb GTPase, which in turn activates mTOR, resulting in the inhibition of autophagy [2, 78]. Akt-mediated phosphorylation of Beclin 1 (ATG6) inhibits autophagy by forming an autophagy-inhibitory complex composed of Beclin 1, 14-3-3 protein, vimentin, and the intermediate filament complex [19]. Akt phosphorylates Unc-51-like autophagy activating kinase 1 (ULK1, ATG1) at Ser774 through insulin signaling [47] and TSC2 [37]. TFEB, a transcriptional regulator of autophagy, is also targeted by Akt to inhibit the induction of autophagy [44, 67].

In contrast to growth factor stimulation—which is known to cause Akt translocation to the plasma membrane—after the induction of autophagy, Akt accumulates in the lysosomes along with Phafin2 [4, 36]. In agreement with this finding, mTORC2, PHLPP1, and Akt are reported to be recruited to lysosomes to regulate chaperone-mediated autophagy [34]. It is plausible that VRK2A, which augments Akt kinase activity and activities of its downstream effectors in the lysosomes, is involved in the regulation of autophagy.

The finding that KD VRK2 failed to interact with Akt supports the idea that the kinase activities of both VRK2 and Akt are important for this interaction. Our BiFC assays suggest that kinase activities of both Akt and VRK2 are crucial not only for lysosomal localization of the VRK2–Akt complex but also for phosphorylation of Akt. The Akt signal in the immunoprecipitation assay (CST, # 9272) after HBSS treatment was lower than that in the untreated cells (Fig. 2a, lower panel). Nevertheless, after immunoprecipitation, VRK2, which interacts with Akt, produced comparable intensity of the signal (Fig. 2a, upper panel). The immobilized Akt antibody (CST, # 9279) used for Akt immunoprecipitation is known to preferentially recognize the phosphorylated form of Akt. The relative intensities of the signals of the interaction between endogenous Akt and VRK2 also favor the idea that VRK2 preferentially interacts with p-Akt.

It is notable that amino acid depletion by HBSS treatment for 8 h, a method that is typically used for the induction of autophagy [59, 79], is sufficient to inhibit the intracellular Akt kinase activity, and we found that levels of phosphorylation of Akt remain high in the lysosomes (Fig. 5a). Phafin2 enhances lysosomal localization of Akt upon the induction of autophagy [36]. It is plausible that additional intracellular factors may be required for increasing Akt phosphorylation in the lysosomes. Alternatively, it is possible that VRK2A preferentially recruits the phosphorylated form of Akt, as suggested by the finding that phosphorylation is required for Akt–VRK2 interactions in coimmunoprecipitation assays or BiFC.

Notably, in line with the above observation, the KD form of VRK2 (in contrast to WT VRK2) failed to maintain the high levels of phosphorylation of Akt at lysosomes in BiFC assays. This is partly due to its inability to associate with Akt, so that it failed to enhance Akt kinase activity. This result suggests that PI3K activity is a prerequisite for the VRK2A-dependent enhancement of Akt kinase activity at the lysosomes. We showed that after the induction of autophagy, which increases lysosomal PtdIns(3)P amounts, Akt accumulated via Phafin2 in a manner that depended on PtdIns(3)P interaction [36, 80].

Vps34-mediated synthesis of PtdIns(3)P is required for the proper sorting of hydrolases from the Golgi complex to the vacuole in order to maintain vacuolar size and membrane homeostasis [81]. Moreover, the essential role of Vps34 in autophagy has been established largely using the pharmacological inhibitor 3-methyladenine (a class III PI3K inhibitor), which suppresses autophagy [82–85]. In agreement with these data, an experiment on Vps34-null cells has revealed that this protein performs an essential function in autophagy in the liver and heart [86]. Activation of general cellular kinase activity of Akt by growth factor stimuli may inhibit the induction of autophagy [29–32]. Notably, however, pan-PI3K inhibitors, such as wortmannin or LY294002, which are known to suppress the production of both PtdIns(3)P and PI(3,4,5)P_3_, inhibit macroautophagy [82]. It is plausible that cross-reactivity of pharmacological inhibition with a different class of PI3K may underlie these complexities [4].

According to the BiFC assay, kinase activities of Akt and VRK2 are important for lysosomal accumulation of protein complexes. Altogether, our results support the following model: lysosomal kinase activity of Akt, as maintained by VRK2 (which preferentially binds to p-Akt), plays a role in the complete process of autophagy, including acidification and activation of hydrolytic enzymes.

We showed that the knockdown of VRK2A decreased the size of lysosomes, and this effect coincided with the diminished nuclear localization of TFEB and impaired lysosomal acidification and activation of cathepsin D after HBSS treatment.

TFEB is known to be phosphorylated at Ser211 by mTOR, which inhibits TFEB activity [68]. Recently, however, Akt was shown to phosphorylate TFEB at Ser467 and to repress TFEB nuclear translocation independently of mTORC1[44]. In this regard, it is noteworthy that 11 phosphorylation sites of TFEB (for different kinases) throughout the mature protein sequence have been identified [45]. It is possible that different combinations of the putative 11 TFEB phosphorylation sites (including both positive and negative combinations of phosphorylation events on the 11 putative phosphorylation sites [45]) present in TFEB and affected by different kinases are involved in the regulation of autophagy.

We used LysoTracker Red DND-99 to determine lysosomal acidification in the process of autophagy induction by HBSS or rapamycin treatment because of the report that mTORC1, a downstream effector of Akt, is critical for control over v-ATPase, the major proton pump on the lysosomal membrane, via lysosomal amino acids through an inside-out mechanism [66]. The results suggest that the VRK2–Akt interaction, which controls functional biogenesis of lysosomes possibly through TFEB, can maintain stable low pH in lysosomes in the process of autophagy in mammalian cells.

We demonstrated that VRK2 is important for the induction of autophagy, for digestion of fluorescent bacteria, and for viral replication. Various host cell factors are required for viral replication [87, 88]. As the end point of endocytosis, lysosomes also act as a safeguard by degrading the macromolecules of a pathogen; thus inhibition of lysosomal function is reported to enhance cellular human immunodeficiency virus infection [70, 71]. Accordingly, the VRK2 knockdown appeared not to affect viral entry but enhanced replication of the influenza virus, when we used PR8 influenza as a model system. Autophagy is thought to participate in the defense mechanisms against intracellular pathogens [7, 71, 89]. Effective activation of the PI3K–Akt pathway through VRK2 at the lysosomes may allow scavengers to control lysosomal acidification during an infectious process [71, 90].

We found that the lysosomal VRK2–Akt complex controls cellular proliferation and survival. There is accumulating evidence for a cross-talk in the regulation of apoptosis and induction of autophagy [9, 10, 42, 91, 92]. The involvement of autophagy in the growth and differentiation of normal and tumorous tissues appears to be complex and context dependent [16, 20, 93]. Attention has turned to autophagy, a survival-promoting pathway for cancer metabolism [9, 13, 94–96], which underlies the mechanisms of initiation and maintenance of various cancers [17–20].

**Fig. 9:**
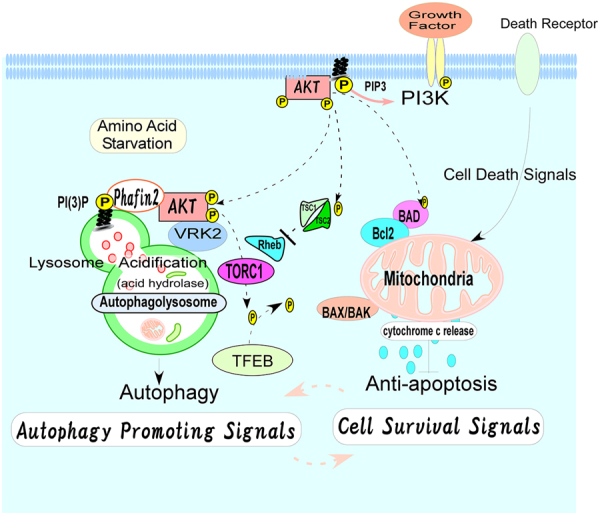
A schematic model of lysosomal Akt-VRK2 as a functional modulator in the regulation of autophagy. In the present study, VRK2 was identified among the molecules that associate with Akt and are enriched after autophagy induction at the lysosomes. We demonstrated that VRK2A facilitates lysosomal accumulation of activated Akt and nuclear localization of TFEB for proper biogenesis of functional lysosomes [44, 67]. It controls the size and acidification of lysosomes and subsequent activation of acid hydrolases to promote the induction of autophagy. Larger amounts of activated Akt accumulated at the lysosomes may serve as a connection between antiapoptotic processes and the induction of autophagy. Although autophagy was originally characterized as a cell survival mechanism in yeast under starvation conditions, it is now thought to be a form of cell death along with the two classical mechanisms, apoptosis and necrosis, in mammalian cells [9–13]. Given that autophagy is involved in various characteristics of initiation and maintenance of cancers [15–20], the VRK2–Akt complex at the lysosomes, which controls the process of autophagy, may be a novel molecular target for cancer therapy via modulation of autophagy [97]

The present study supports the paradigm that lysosomal VRK2 serves as a functional regulator of autophagy via the recruitment of activated Akt to lysosomes to control the proper biogenesis of functional lysosomes, which could be a novel molecular target for cancer therapy [97] (Fig. 9).

## Materials and methods

### Purification and TOF/MS analysis of the Akt interacting molecules at lysosome

FLAG- Akt2 and HA-Phafin2 were transformed into 293T cells, treated with HBSS for 4 h (Sup. 1a-b). The cells were harvested, rinsed twice with ice-cold phosphate-buffered saline. The lysosomal-enriched fraction was purified using the Lysosome Isolation Kit (LYSISO1, Sigma), precleaned with ProA/ProG beads, immunoprecipitated with FLAG-M2 beads (Sigma), and subjected to MS/MS analysis by LXQ (Thermo Fisher) equipped with nano-flow LC Magic (Michrom Bioresources, Inc.) [36]. The spectra for potential peptide that are enriched after autophagy induction were determined.

### Construction of VRK2 and related expression vectors

Human VRK2 cDNA was purchased from Origene (RC206522) and the constructs were generated. The primers and the vectors for PCR are listed in supplemental information.

### Coimmunoprecipitation

Coimmunoprecipitation experiments were performed as described [76, 98, 99]. For endogenous interaction, HT 1080 ells (ATCC) were cultured in the presence of chloroquine (25 μM, Sigma) before rapamycin (10 μM) or HBSS treatment for 4 h. The cells were harvested, lysed with modified RIPA buffer [100] with proteinase and phosphatase inhibitors with chloroquine for Akt immunoprecipitation, or lysed with RIPA buffer [54] with dithiobis [succinimidylpropionate] (100 μM, Pierce). The cells were immunoprecipitated with anti-Akt (1G1) mAb-beads (#9279, CST) [76, 98, 99] with modified RIPA buffer [100], RIPA buffer [54], or Brij97 cell lysis buffer [76, 98].

### Colocalization experiment using a confocal microscopy

HeLa cells, A549, or J774.1 (ATCC) cultured to sub-confluency, were harvested, fixed with 3.7% formaldehyde, immunostained with the indicated antibodies and DAPI (4’,6-diamidino-2-phenylindole, blue, Sigma), and examined using a confocal microscopy (FLUOVIEW FV-1000, Olympus) [76, 98, 99].

### Bimolecular fluorescence complementation

BiFC analysis was essentially described previously [36, 101] using pCMV-VN-Empty, pCMV-VC-Empty, pCMV-VN-VRK2A, pCMV-VC-Akt2, pCMV-VN-VRK2A-Kinase Dead, or pCMV-VC-Akt2-T308A/S473A. Transfected cells were immunostained with the indicated antibodies combined with DAPI staining, mounted on Fluoromount/Plus (VWR, 95041-480), or stained with Lysotracker Red DND-99 (Thermo Fisher) and analyzed using confocal laser microscope [98]. Relative intensities of these signals (BiFC, LAMP2, and aAKtS473), the quantification of white signals/red signal (lysosome) were calculated and shown as bar graph with statistics.

### Lentiviral shRNA and siRNA experiments with immunostaining

shRNA lentivirus vectors were generated in pLKO.1 (Addgene) with VRK2 shRNAs and scrambled shRNA or enhanced green fluorescent protein (EGFP) shRNA as a control. VRK2A (WT and KD) in lentiviral vector were generated in pCSII-EF1α-FLAG-VRK2A (WT or KD)-IRES2-Blasticidin [102–104] using stable HeLa or J774.1 cells. Phafin2 Si RNA was conducted as described [36]. Cells were transduced with the indicated VRK2 shRNAs, harvested, incubated with HBSS, immunostained, and analyzed using confocal microscopy.

### Transmission electron microscope

HeLa cells (ATCC) transfected with VRK2-shRNA (plus pBluescript or shRNA -07-resistant VRK2A) were treated with HBSS for 8 h to induce autophagy. TEM was conducted using the electron microscope (H-7100; Hitachi) [36, 99].

### Fluorescent *E. coli* experiment

J774.1 cells (ATCC) stably transduced with lentiviral VRK2 shRNA (VRK2-784 for mouse VRK2 or EGFP as a control) were analyzed as described [36] using a confocal microscope (FLUOVIEW FV1000-D, Olympus) with Lysotracker Red DND-99 staining.

### Cathepsin D assay

Cathepsin D assays were performed using the SensoLyte^TM^ Cathepsin D Assay Kit (ANASPEC, Inc. CA), using the indicated VRK2 shRNA-transduced HeLa cells. The data shown are normalized by protein concentration measured by Bio-Rad Protein Assay.

### Influenza virus plaque assays

A549 cells were infected with the PR8 virus, and the plaque assays were conducted using MDCK cells as described previously [105].

### Proliferation assays

Proliferation assays were performed using stably VRK2 shRNA (designed as #02 or #07) expressing 293T or Hela cells as described [98].

### Chimeric Phafin2-Akt experiments

Phafin2-chimeric Akt (WT/AA/DD, see supplemental information) were generated and transfected into Hela cells to induce autophagy. Lysosomal localization was determined by LAMP2 staining, and the induction of autophagy was assessed by LC3 and p62.

### MTP measurement

The Rh 123 fluorescence intensity for MTP were analyzed as described [76, 98].

### Statistical analysis

Statistical analyses were performed by Student’s *t*-test.

## Electronic supplementary material

Supplemental data and legend

Supplemental Fig 1

Supplemental Fig 2

Supplemental Fig 3

Supplemental Fig 4

Supplemental Fig 5

Supplemental Fig 6

Supplemtal Fig 7
