# Supplementary material for: Functional characterization of lysosomal interaction of Akt with VRK2
Source: Oncogene. 2018 Jun 5;37(40):5367–86. doi: 10.1038/s41388-018-0330-0 (PMC6172193; doi:10.1038/s41388-018-0330-0)
Supplement: Supplementary file 1 — Supplemental data and legend [file 41388_2018_330_MOESM1_ESM.docx]

**Supplemental data**

**Supplemental data 1　(Figure 1 related)**

**Purification and TOF/MS analysis of the Akt interacting molecules at lysosome Supplemental data 1a**

We searched for the molecules that interact with Akt and are enriched at the lysosomes after induction of autophagy ^6^. For this purpose, Akt–Phafin2-transfected 293T cells were treated with HBSS to induce autophagy, and the lysosome fraction was then enriched as described and immunoprecipitated using anti-FLAG beads for TOF/MS analysis (**Supplemental data 1a**-**b**). The purity of the lysosomal enrichment was validated by EM previously ^6^. Among the molecules that interact with Akt after induction of autophagy, the VRK family kinases, a serine thereonine kinase in the human kinome was identified. Coomassie Brilliant Blue stained SDS-PAGE of the anti-FLAG (Akt) immune complexes before and after HBSS treatment for MS analysis was shown. Please note strong band of both Akt and Phafin2 were present on the SDS-PAGE gel with dominant bands (arrowheads).

**Supplemental data 2 (Figure 1 & 2 related)**

**Supplemental data 2a.**Amino acid sequence alignments of human VRK family kinases (VRK1, VRK2, and VRK3) identified were shown. Kinase domain is shown by red box. Blue box indicates transmembrane domain present in VRK2A ^4, 7, 8, 10^.

**Supplemental data 2b.** Structure of VRK2A and VRK2B

Structure of VRK2A and VRK2B are shown schematically.

Three isoforms of the VRK family of kinase (VRK1, VRK2, and VRK3) are present in the human genome, and show 22–44% sequence homology at the amino acid level. Among the three isoforms, both VRK1 and VRK2, but not VRK3, are catalytically active. Moreover, two alternative splice variants of VRK2 (VRK2A and VRK2B) are present in mammalian cells; VRK2A, but not VRK2B, harbors a transmembrane domain at its C-terminus for endosomal and/or lysosomal localization.

***mTOR activity and S6K kinase activity is stable in VRK2 shRNA Cells*** (Supplemental data 3, Figure 4 & 5 related)

Supple 3a. In order to evaluate the role of VRK2 for the mTOR activity, we have examined the total and phospho-mTOR immunostaining in HeLa cells (ATCC) and the levels of phospho mTOR immunoblot in the cells stably transduced shRNA for VRK2 before and after the induction of autophagy. Inhibition of VRK2 expression by two different shRNAs (shRNA-07 or shRNA-02), which drastically inhibited the expression of endogenous VRK2 (see Fig. 4b top panel lines 1, 3, and 5), did not affect expression of mTOR or p-mTOR by confocal microscopy.

Supple 3b-d. The levels of phospho-mTORand phosphor-S6K at the lysosome was examined in the cells transfected with VRK2 shRNA after the treatment of HBSS or Rapamycin to induce autophagy by immunoblot (Supple 3b). No decrease in the p-mTOR/total mTOR ratio or p-S6K/total S6K ratio was induced by HBSS treatment of the cells transfected with VRK2-02 and VRK2-07 shRNAs compared to the scrambled control (Supple 3c-d, n = 3, p > 0.20). Please note that there was no significant change in the actual value of the p-mTOR/total mTOR ratio (or p-S6K/total S6K ratio) after induction of autophagy by the VRK2 knockdown.

Supple 3e. Increasing amount of VRK2A (lanes 1-4, top panel) enhances Akt kinase activity determined by anti-phospho-Ser473antibody blotting (Cell Signaling Technology , #400, lanes 1-4, fourth panel from the top). However,

Both mTOR or phospho-mTOR did not affected by VRK2 transfected cells determined by immunoblot .

The observation supported that impaired inhibition of autophagy is not through the inhibition of mTOR. The expression of VRK2 by shRNA were verified by immunostaining (**Figure 5b**).

**Method. (a** and **b).** HeLa cells (ATCC) stably transduced with indicated shRNA (VRK2 #2 and #7) or control (scrambled shRNA) were washed twice with HBSS, then incubated with HBSS for additional 8 hours or 10μM rapamycin for 4 hours to induce autophagy. The cells were immunostained with indicated antibodies (Total mTOR, Cell Signaling Technology #2983, Phospho-mTOR, Cell Signaling Technology #5536, and LAMP2, Abcam, ab25631) together with DAPI (4’, 6-diamidino-2-phenylindole, blue, Sigma) and observed using confocal microscopy (FLUOVIEW FV1000-D, Olympus). For the immunoblot, the cells were harvested, lysed with cell lysis buffer (modified RIPA buffer ^11, 13^) in the presence of phosphatase inhibitor, and resolved onto SDS gel and immunoblotted by indicated antibodies, and detected by ECL. (c) Total amounts of 0~2.4μg/dish of Flag-VRK2A were transfected into HT1080 cells (ATCC) using Lipofectamine LTX (Invitrogen). Total amount of plasmid transfection (2.4μg per well) were adjusted by adding back p-Bluscript to keep constant. Forty eight hours after transfection, the cells were harvested and lysed with modified RIPA buffer ^11, 13^ and 20μg of total protein were resolved onto SDS-PAGE and immunoblotted using the indicated antibodies and detected by ECL.

***Immunostaining of VRK2 after induction of autophagy* (Supplemental data 4)**

**(Fig 5 related).**

Immunofluorescent study revealed that VRK2 were accumulated at the lysosome after induction of autophagy as determined by immunostaining. Further, VRK2 and phospho-Akt were co-stained after induction of autophagy.

**Methods:** HeLa cells were cultured in DMEM with 10% FBS with Penicillin/ Streptomycin (P/S) at sub-confluency condition. The cells were cultured in DMEM with 10% FBS with P/S or HBSS treatment to deplete serum to induce autophagy, fixed with 3.7% formaldehyde, and immunostained with indicated antibodies (Anti-VRK2,Anti-Phospho-Akt CST # 4051, and Anti-LAMP2 antibody, Abcam, ab25631) and DAPI (4',6-diamidino-2-phenylindole, blue, Sigma), and examined using a confocal microscopy (FLUOVIEW FV-1000, Olympus) as described ^12, 13^.

**Inhibition of Phafin2 by siRNA abolish lysosomal localization of VRK2 after induction of autophagy (Supplemental data 5).**

1. Phafin2-siRNA treatment resulted in the reduction of lysosomal accumulation of VRK2 after induction of autophagy by rapamycin treatment (10μM) or HBSS treatment for 4 hours to induce autophagy using J774.1 murine macrophage cells.
2. Quantitation of co-localization (signals/pixels) of VRK2 and lysosome (anti-LAMP2) were shown in bar graph with statistical differences (n=30).
3. Phafin2 siRNA inhibited the expression of endogenous Phafin2 in J774.1 cell lines.

**Method:** J774.1 cell lines (murine macrophage cells, ATCC) were transfected with siRNA specific for Phafin2 [MSS231003 (Phafin2-2): Mouse Stealth Select RNAi: Plekhf2 Stealth Select RNAi™ 3 siRNA, Invitrogen] or firefly luciferase as control (Wako Nippon GENE) using CUY21 Pro-vitro (NEPAGENE Co. Ltd) as described^6^. Forty eight hours after transfection, the cells were treated with HBSS (Hank’s Balanced Salt Solution, Gibco, 14025-092) or rapamycin (10μM) for 4 hours, fixed, and immunostained with anti-LAMP2 (Anti-LAMP2 antibody, Abcam, ab25631), anti-VRK2 (Anti-VRK2 antibody, ab58052) along with DAPI (4',6-diamidino-2-phenylindole, blue, Sigma), and analyzed using confocal microscopy (FLUOVIEW FV-1000, Olympus). For immunoblot, cells were lysed and resolved onto SDS-PAGE and immunoblot using anti-Phafin2 antibody (home made) or indicated antibodies and detected by ECL (panel c)^6^.

**The role of TFEB (Supplemental data 6a-c)**

We next examine the expression pattern of TFEB as possible target of VRK2 for controlling the biogenesis of functional lysosome. TFEB, a master regulator for biogenesis of lysosome for induction of autophagy, is known to be localized at the cytosol as nonphosphorylated form.

After HBSS treatment to induce autophagy, TFEB is localized at both cytosol and nucleus TFEB in control shRNA transfected cells (Supplemental data 6a). In contrast, TFEB predominantly localized at the cytosol in both control and VRK2 knock down cells (both #2 and #7 shRNA for VRK2) before induction of autophagy. After HBSS treatment to induce autophagy, TFEB localized dominantly in the cytosol in VRK2 knock out cells (both #2 and #7 shRNA for VRK2 transduced cells) compared to the scrambled shRNA transfected cells (Supplemental data 6a and b for quantification from three independent experiments). Please note that TFEB localized both cytosol and nucleus in control cells (top right side panels).

Since phospho specific antibodies for TFEB are not readily available from commercial sources, we examined the migration shift of TFEB phosphorylation on Mn^2+^ Phos-tag SDS PAGE (Phos-tag^TM^ AAL-107, Nard Institute, Japan) based on the mobility shift by the levels of TFEB phosphorylation by immunoblot using anti-TFEB antibody (Cell Signaling Technology #4240)^3, 15^(supplemental data 6c, top panel). After HBSS treatment, we observe that TFEB migrated faster in control/scrambled ShRNA transfected cells (supplemental data 7c, lane 4), presumably due to lower levels of phosphorylation compared to the VRK2 knock down cells (supplemental data 7c, lane 2 and lane 6, #2 and #7 VRK2 shRNA cells, respectively).

**Method: TFEB analysis (Sup 6a-c)** HeLa cells (ATCC) transduced with indicated shRNA (VRK2 #02 and #07) or control (scrambled shRNA) were washed twice with HBSS, and then incubated with HBSS for additional 8 hours to induce autophagy. For confocal microscopy, these cells were immunostained with anti-TFEB antibody (Cell Signaling Technology #4240) together with DAPI (4’, 6-diamidino-2-phenylindole, blue, Sigma) and observed using confocal microscopy (FLUOVIEW FV1000-D, Olympus).

For immunoblot, cells were lysed with RIPA buffer (50mM Hepes pH 7.4, 150mM NaCl, 1.5mM MgCl_2_, 10mM NaF, 10% glycerol, 1% Triton X-100, 0.1% SDS^2^) plus proteinase inhibitors (AEBSF, Leupeptin) and phosphatase inhibitors (Na_3_VO_4_, NaF)^5, 12, 14^ in the absence of EDTA. The resultant samples were resolved onto 6% SDS-PAGE (supplemental data 6c, middle panel) or Mn^2+^ Phos-tag SDS PAGE (Phos-tag^TM^ AAL-107, Nard Institute, Japan, supplemental data 6c top panel) and immunoblotted using anti-TFEB antibody (Cell Signaling Technology #4240) or anti-α tubulin (Sigma) and detected by ECL according to the manufacturer’s instruction.

**Lysotracker staining (Supplemental data 7a) (Figure 7 related)**

Lysotracker (Lysotracker Red DND-99, L-7528, Thermo Fisher) was used to determine the lysosomal acidification after HBSS (middle panels) or rapamycin treatment (right side panels) to induce autophagy according to the manufacture’s instruction. Inhibition of VRK2 expression was verified by immunoblotting (see Figure 5b, top panel).

**VRK2A enhances natural cell growth (Figure 8 related)** **(Supplemental data 7b)**

Wild type VRK2A enhances natural cell growth on 293T cells.

**Method:** Proliferation assays were analyzed by xCelligence system (Real Time Cell Analyzer, Roche) using 293T cells transfected with 2 μg/6 well plates of WT Flag-VRK2A using PEI^12^.

***Antibodies used in the study***

Anti-phospho-Akt (Ser473) antibody (#9271, Cell Signaling Technology,CST), anti-phospho-Akt (Ser473) (D9E) XP® rabbit mAb (#4060, CST), anti-phospho-Akt (#4051 CST), anti-Akt antibody (#9272, CST), anti-Akt (pan) (40D4) mouse mAb (#2920, CST), anti-HA mAb (12CA5, Roche), anti-FLAG-M2 antibody (F3165, Sigma), anti-GFP pAb (MBL, 598), anti-Mouse IgG1 antibody (X0931, Dako), anti-HA-HRP High Affinity (3F10, 2013819, Roche), anti-FLAG-M2-HRP conjugate (A8529, Sigma), anti-GFP-HRP-DirecT (MBL, 598-7), anti-LC3A/B (D3U4C) XP® rabbit mAb (#12741, CST), anti-LC3 antibody (MBL, PM036), Anti-SQSTM1 / p62 (D5E2) rabbit monoclonal antibody (#8025), monoclonal anti-alpha-Tubulin (clone DM1A, T9026, Sigma), Anti-LAMP1 (D2D11) antibody(#9091,CST),anti-LAMP2 (H4B4) mAb (ab 25631), Total mTOR (#2983,CST) and Phospho-mTOR (#5536, CST), total p70S6 kinase (#9202, CST), phospho-p70S6 kinase (T389) #9234S), and anti-TFEB antibody (#4240, CST).

**List of PCR primers for plasmid construction.**

Plasmid vector (pCMV6-myc-DDK) containing a full cDNA fragment of human VRK2 was purchased from Origene (RC206522).

Full length VRK2A or VRK2B fragments were generated by PCR amplification using pair of primers (HindIII and BamHI sites for subcloning are underlined) HD946: Sense, 5’-ATCATCAAGCTTATGCCACCAAAAAGAAATG-3’, HD947: Anti-sense; 5’-ATCATCGGATCCTCAGAGAAAAAATAAAGC-3’) and VRK2B (HD946: Sense; 5’-ATCATCAAGCTTATGCCACCAAAAAGAAATG-3’ HD948: Anti-sense; 5’-ATCATCGGATCCCTACCTAAACTGAGCTGCTTC-3’) with human VRK2A as a template, and subcloned into pFLAG-CMV2-VRK2A, pFLAG-CMV2-VRK2B, pEGFP-C2-VRK2A, or pEGFP-C2-VRK2B.

Subfragment of VRK2A in pFLAG-CMV2 (Sigma) were generated using the following primer pairs were underlined) with PCR or QuikChange Site-Directed Mutagenesis Kit (Agilent Technologies).

VRK2A-C-term (Hind III/BamHI sites were underlined)

HD998: Sense: 5’-ATCATCAAGCTTATGTTAGGACCACTGGACTTTTCCAC-3’

HD948: Anti-sense: 5’-ATCATCGGATCCCTACCTAAACTGAGCTGCTTC-3

VRK2A (N-terminal VRK2A, artificially created stop codon was underlined)

HD999: Sense, 5’-CTCATGGAATACCTTAAGGACCACTGGACTT-3’

HD1000: Anti-sense, 5’-AAGTCCAGTGGTCCTTAAGGTATTCCATGAG-3’

VRK2A or VRK2B kinase-dead (K61A/K169E:ATP binding site mutant and kinase active site mutant) were generated by QuikChange Site-Directed Mutagenesis Kit, (Agilent Technologies) using the following primers with mutated codons are underlined (single lines for the mutated codon, and double lines for the substituted nucleotides): K169E (HD1003: Sense, 5’-GTTCATGGTGATGTAGAAGCAGCAAATCTAC-3’ and HD1004: Anti-sense, 5’-GTAGATTTGCTGCTTCTACATCACCATGAAC-3’) and K61A (HD1001: Sense, 5’-CAAGACATGTA/GTAGCAGTGGAATATCAA/G-3’ and HD1002: Anti-sense, 5’-CTTGATATTCCACTGCTACTACATGTCTTG-3’) and subcloned into pEGFP-C2 (Clontech) or BiFC vector (pCMV-VN-FLAG).

shRNA resistant VRK2 expression construct VRK2AshRNA^r^ was generated using the following primer pairs (mutated nucleotide are underlined) with QuikChange Site-Directed Mutagenesis Kit (Agilent Technologies).

HD1091: shRNA-07 resistant VRK2 Sense

5’- CGA ATG TTG GAC GTC CTA GAG TAT ATA CAT -3’

HD1092: shRNA-07 resistant VRK2 Anti-Sense

5’- ATG TAT ATA CTC TAG GAC GTC CAA CAT TCG -3’

The DNA sequences of the final contract were verified by nucleotide sequences.

**VRK2A (WT and KD) in lentiviral vector**

VRK2A (WT and KD) in lentiviral vector were generated in pCSII-EF1α-FLAG-VRK2A (WT or KD)-IRES2-Blasticidin ^1^.

**BiFC constructs [pCMV-Venus-N-terminal-Tag2 (pCMV-VN-FLAG) and pCMV-Venus-C-terminal-Tag2 (pCMV-VC-FLAG)]**

**Method:** Venus-N-terminal fragment (VN, 1-172 amino acids) was generated by PCR amplification using the following pairs of primers (HD992: Sense; 5’-ATCATCCCACCGCGATGGTGAG CAAGGGC-3’ and HD993: Anti-sense: 5’-ATCATCGCGGCCGCCTCGATGTTGTG GCGGATCTTG-3’, BstXI and NotI sites for subcloning purpose were underlined). Venus-C-term (VC, 173-238 amino acids) was generated by PCR amplification using the following pairs of primers, HD994: Sense: 5’-ATCATCCCACCGCGATGGACGGCGGCGTGCAGCTCG-3’, HD995: Anti-sense:5’-ATCATCGCGGCCGCCTTGTACAGCTCGTCCATGCC-3’) and subcloned into pCMV-Tag2B (Stratagene).

**Lentiviral VRK2 shRNA expression vectors**

**Method:** Lentivirus vectors for VRK2 shRNA were generated in pLKO.1 puro vector (Addgene #10878) with scrambled shRNA (Addgene, Plasmid #1864) as a control.

VRK2 targeting shRNAs were designed by the RNA Consortium for the following targeting VRK2 sequences (VRK2 shRNA-02:

5’-GATCTCCATCTTGGTATAAAT-3’) and VRK2 shRNA-07:5’-GTTGGATGTACTGGAATATAT-3’). EGFP shRNA was purchase from Addgene (pLKO.1 GFP shRNA, #30323).

The primer pairs used for VRK2 shRNA are listed below with additional nucleotide sequences for subcloning {AgeI (A/CCGGT) and EcoRI (G/AATTC)} are underlined.

**VRK2 shRNA-02:**

Forward Oligo (HD1022):

5’-CCGG-GATCTCCATCTTGGTATAAAT-CTCGAG-ATTTATACCAAGAT GGAGATC-TTTTTG-3’

Reversed Oligo (HD1023):

5’-AATT-CAAAAA-GATCTCCATCTTGGTATAAAT-CTCGAG-ATTTATACC AAGATGGAGATC-3’

**VRK2 shRNA-07:**

Forward Oligo (HD1024):

5’-CCGG-GTTGGATGTACTGGAATATAT-CTCGAG-ATATATTCCAGTACAT CCAAC-TTTTTG-3’

Reversed Oligo (HD1025):

5’-AATT-CAAAAA-GTTGGATGTACTGGAATATAT-CTCGAG-ATATATTC CAGTA CATCCAAC-3’

**Mouse VRK2 shRNA-784:**

Target sequence: 5’- GTACTTGATGTATGTTCATAA-3’

Forward Oligo (HD1116) :

5’-CCGG-GTACTTGATGTATGTTCATAA-CTCGAG-TTATGAACATACATCAAGTAC-TTTTTG-3’

Reversed Oligo (HD1117) :

5’-AATT-CAAAAA-GTACTTGATGTATGTTCATAA-CTCGAG-TTATGAACATACATCAAGTAC-3’

**Phafin2-fused Akt2 (WT, T308A/S473A or T308D/S473D) in pECFP-C1 constructs**

First, (T308D/S473D) were introduced into human Akt2 cDNA by Quikchange using the following pairs of primers

Primers for T308D: (HD535: Sense,5’-GCCACCATGAAAGACTT CTGTGGGACC-3’HD536: Anti-sense,5’-GGTCCCACAGAAG TCTTTCATGGTGGC-3’). Primers for S473D (HD537: Sense, 5’-CTTCCCCCAGTTCG ACTACTCGGCCAGC-3’ and HD538: Anti-sense, 5’-GCTGGCCGAGTAGTCGAACTGGGGGAAG-3’)

Chimeric constructs of Phafin2-Akt2-CT (WT, T308A/S473A^12^ or T308D/S473D) were generated by inverse PCR methods using pFLAG-CMV2-Phafin2 as a template ^6^.

First, human Akt2 C-terminal kinase domain (CT) were amplified by the following pairs of primers with PstI and BamHI (underlined) for cloning purpose.

HD766: Sense 5’-ATCATCCTGCAGTTCGACTATCTCAAACTCCTTGGC-3’

HD767: Anti-sense 5’-ATCATCGGATCCTCACTCGCGGATGCTGGCCGA-3’

Phafin2 with pFLAG-CMV2 vector were amplified with following pairs of primers with PstI and BamHI (underlined) for subcloning purpose using pFLAG-CMV2-Phafin2 as a template^6^.(HD768: Anti-sense 5’-ATCATCCTGCAGGTCACTG CTATCATCATCGTCATC-3’, HD769: Sense 5’- ATCATCGGATCCCGGGTGGCA TCCCTG-3’).

The resulted chimeric Phafin2-Akt2-CT (WT, T308A/S473A or T308D/S473D) or Akt2-WT were subcloned into pECFP-C1 vectors using following primer pairs with EcoRI and BamHI for subcloning purpose (underlined) (HD815: Sense 5’-ATCATC GAATTCA ATGGTGGATCGCTTGGCAAAC-3’ and HD767: Anti-sense 5’- ATCATCGGATC CTCACTCGCGGATGCTGGCCGA -3’) for chimeric constructs or (HD887: Sense 5’-ATCGAATTCAATGAATGAGGTGTC TGTC ATCAAAG-3’: HD151: Anti-sense 5’-ATCGGATCCTCACTCGC GGATGCTGGCC-3’) for Akt2-WT.

***References***

1 Bai Y, Soda Y, Izawa K, Tanabe T, Kang X, Tojo A *et al*. Effective transduction and stable transgene expression in human blood cells by a third-generation lentiviral vector. Gene Ther 2003; 10: 1446-1457.

2 Blanco S, Klimcakova L, Vega FM, Lazo PA. The subcellular localization of vaccinia-related kinase-2 (VRK2) isoforms determines their different effect on p53 stability in tumour cell lines. The FEBS journal 2006; 273: 2487-2504.

3 Chen Y, Dorn GW, 2nd. PINK1-phosphorylated mitofusin 2 is a Parkin receptor for culling damaged mitochondria. Science 2013; 340: 471-475.

4 Klerkx EP, Lazo PA, Askjaer P. Emerging biological functions of the vaccinia-related kinase (VRK) family. Histol Histopathol 2009; 24: 749-759.

5 Laine J, Kunstle G, Obata T, Sha M, Noguchi M. The protooncogene TCL1 is an Akt kinase coactivator. Mol Cell 2000; 6: 395-407.

6 Matsuda-Lennikov M, Suizu F, Hirata N, Hashimoto M, Kimura K, Nagamine T *et al*. Lysosomal interaction of akt with phafin2: a critical step in the induction of autophagy. PloS one 2014; 9: e79795.

7 Nezu J, Oku A, Jones MH, Shimane M. Identification of two novel human putative serine/threonine kinases, VRK1 and VRK2, with structural similarity to vaccinia virus B1R kinase. Genomics 1997; 45: 327-331.

8 Nichols RJ, Traktman P. Characterization of three paralogous members of the Mammalian vaccinia related kinase family. J Biol Chem 2004; 279: 7934-7946.

9 Palmieri M, Pal R, Nelvagal HR, Lotfi P, Stinnett GR, Seymour ML *et al*. mTORC1-independent TFEB activation via Akt inhibition promotes cellular clearance in neurodegenerative storage diseases. Nature communications 2017; 8: 14338.

10 Sanz-García. M, Valbuena A, Lopez-Sanches I, Blanco S, Fernandez IF, Vazquez-Cedera M *et al*. Vaccinia-related kinase (VRK) signaling in cell and tumor biology. Emerging pathways in Tumor Biology 2010: 135-156.

11 Singla V, Romaguera-Ros M, Garcia-Verdugo JM, Reiter JF. Ofd1, a human disease gene, regulates the length and distal structure of centrioles. Developmental cell 2010; 18: 410-424.

12 Suizu F, Hiramuki Y, Okumura F, Matsuda M, Okumura AJ, Hirata N *et al*. The E3 ligase TTC3 facilitates ubiquitination and degradation of phosphorylated Akt. Developmental cell 2009; 17: 800-810.

13 Suizu F, Hirata N, Kimura K, Edamura T, Tanaka T, Ishigaki S *et al*. Phosphorylation-dependent Akt-Inversin interaction at the basal body of primary cilia. EMBO J 2016.

14 Suizu F, Hirata N, Kimura K, Edamura T, Tanaka T, Ishigaki S *et al*. Phosphorylation-dependent Akt-Inversin interaction at the basal body of primary cilia. EMBO J 2016; 35: 1346-1363.

15 Sumara G, Formentini I, Collins S, Sumara I, Windak R, Bodenmiller B *et al*. Regulation of PKD by the MAPK p38delta in insulin secretion and glucose homeostasis. Cell 2009; 136: 235-248.

16 Vazquez-Cedeira M, Barcia-Sanjurjo I, Sanz-Garcia M, Barcia R, Lazo PA. Differential inhibitor sensitivity between human kinases VRK1 and VRK2. PloS one 2011; 6: e23235.
