# Supplementary figures and images for: Functional characterization of lysosomal interaction of Akt with VRK2
Source: Oncogene. 2018 Jun 5;37(40):5367–86. doi: 10.1038/s41388-018-0330-0 (PMC6172193; doi:10.1038/s41388-018-0330-0)

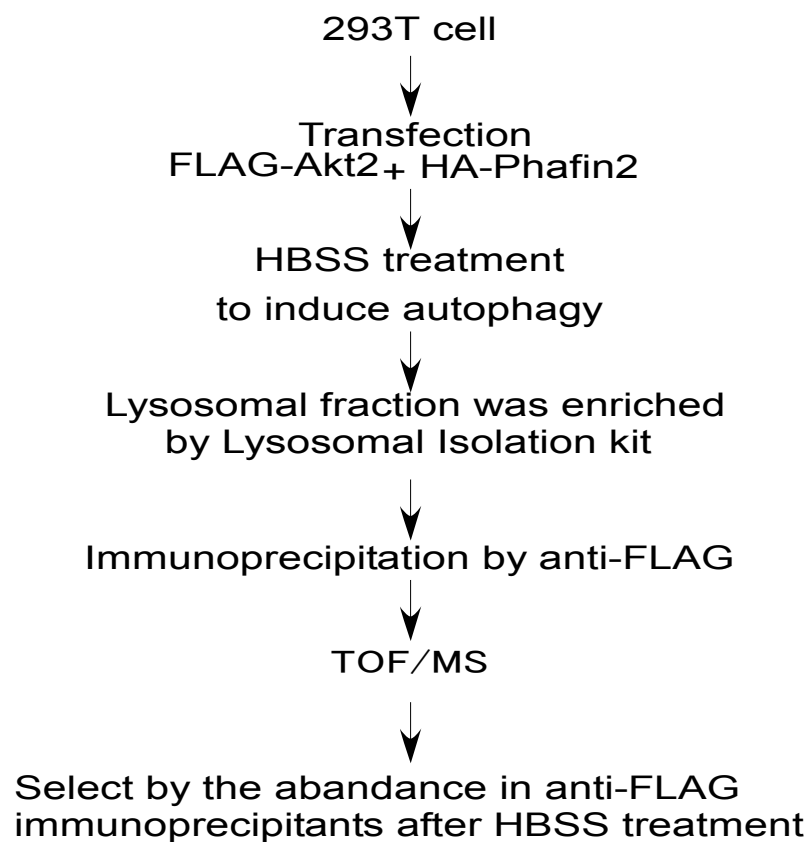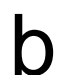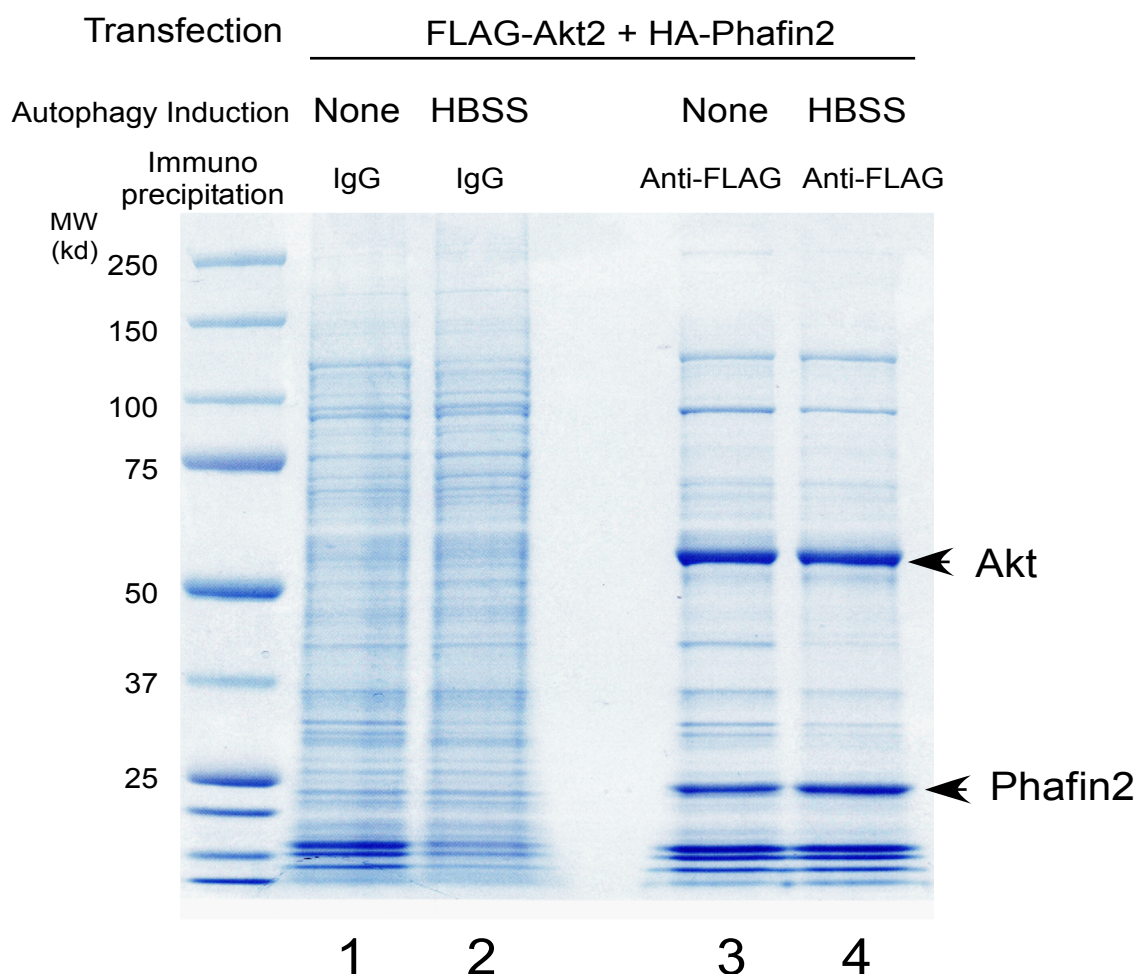

Supplement: Supplementary file 2 — Supplemental Fig 1 [file 41388_2018_330_MOESM2_ESM.pdf]

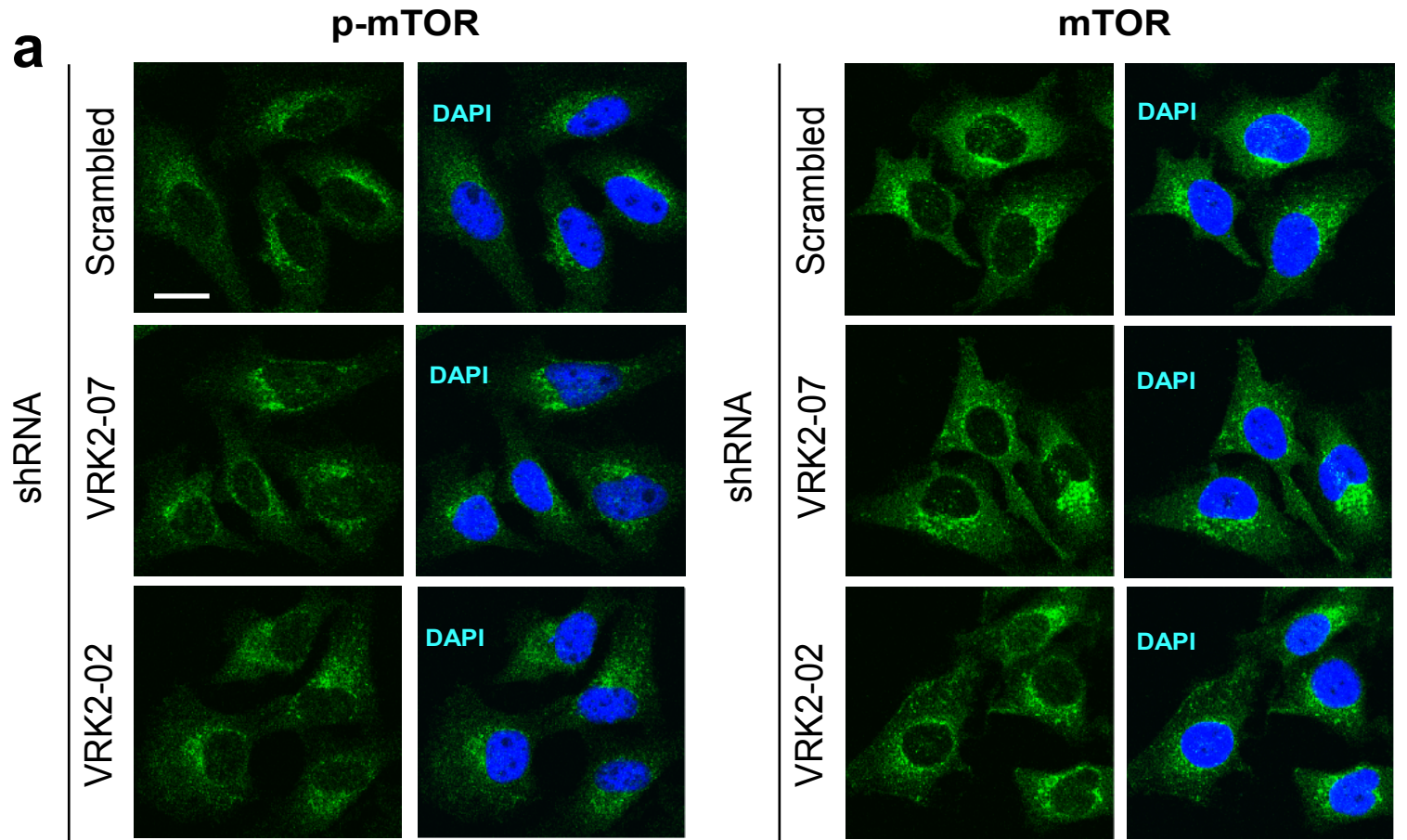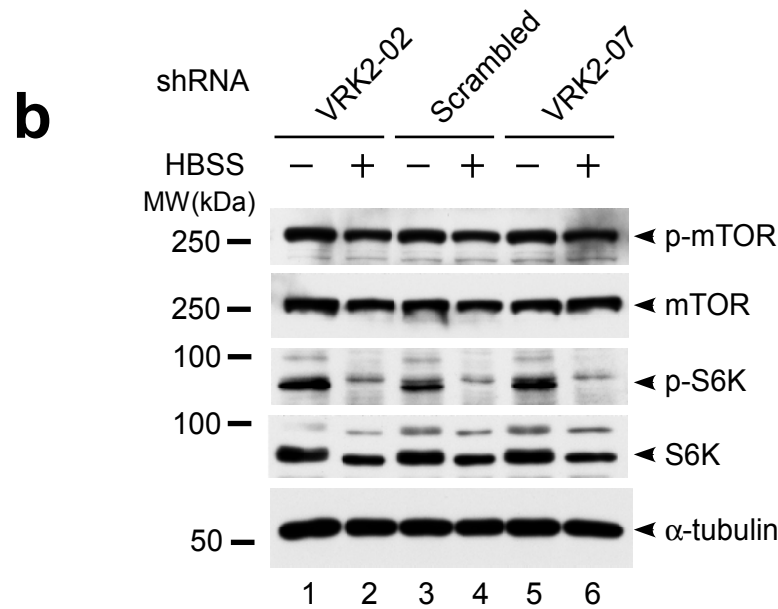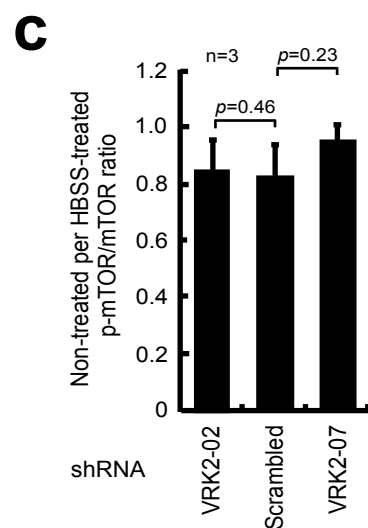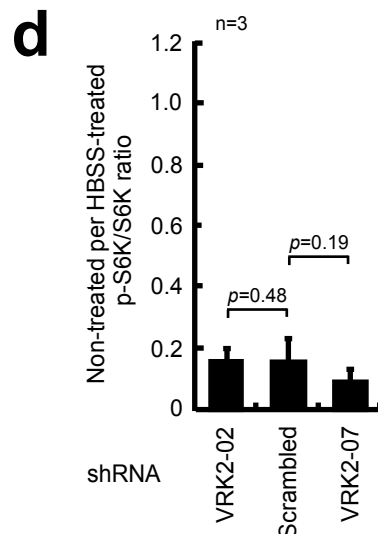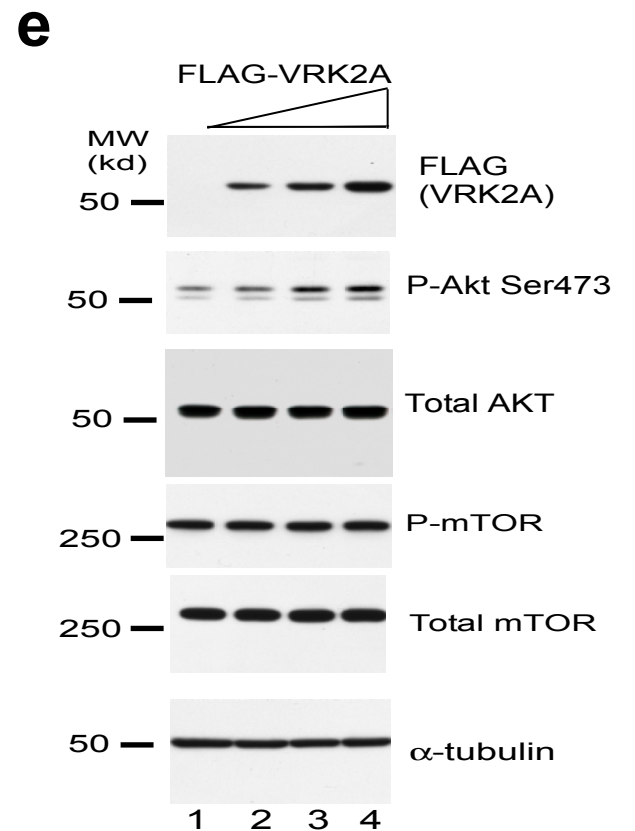

Supplement: Supplementary file 4 — Supplemental Fig 3 [file 41388_2018_330_MOESM4_ESM.pdf]

# Supplemental data 4

Hirata et al.

HBSS

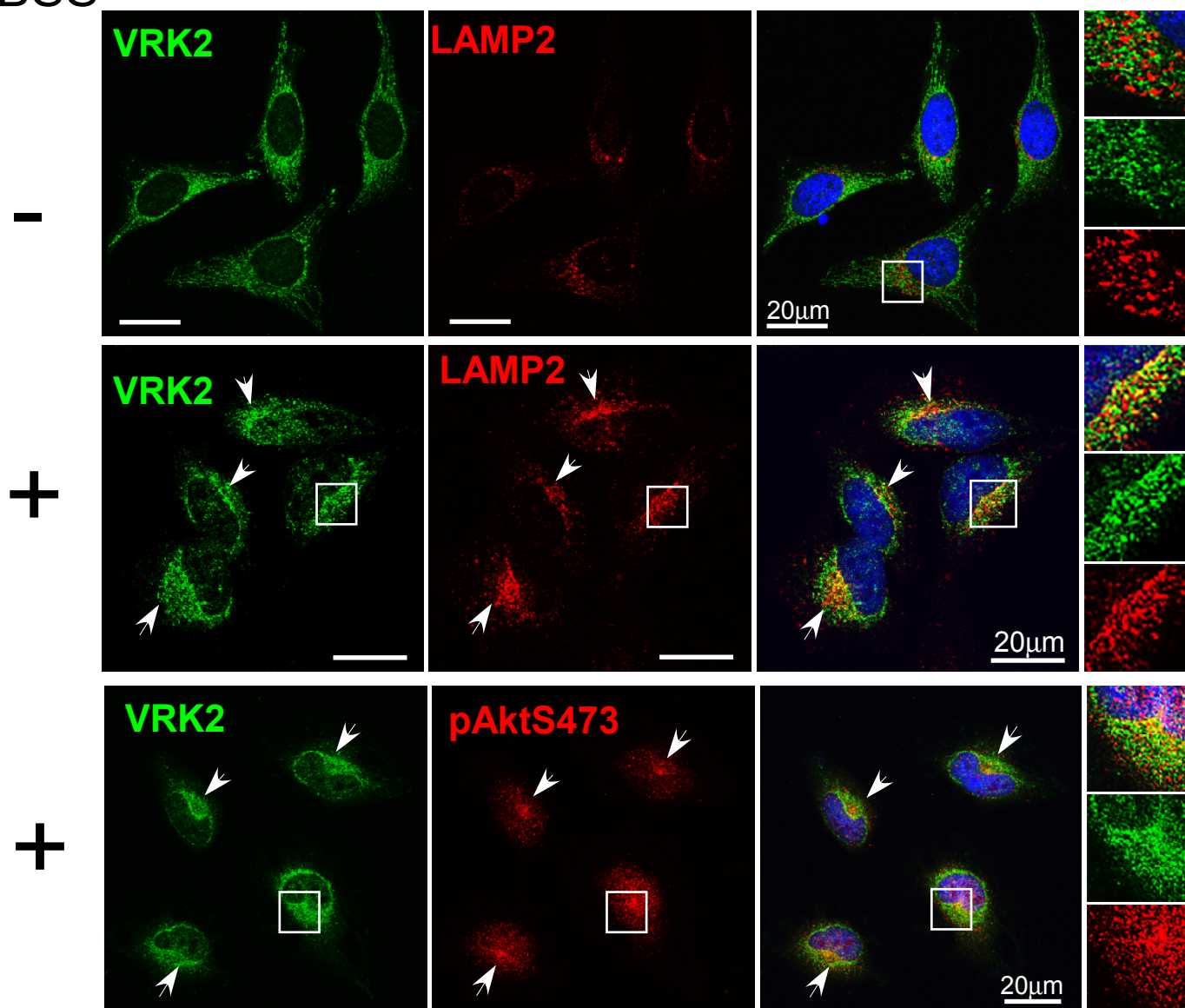

Supplement: Supplementary file 5 — Supplemental Fig 4 [file 41388_2018_330_MOESM5_ESM.pdf]

a

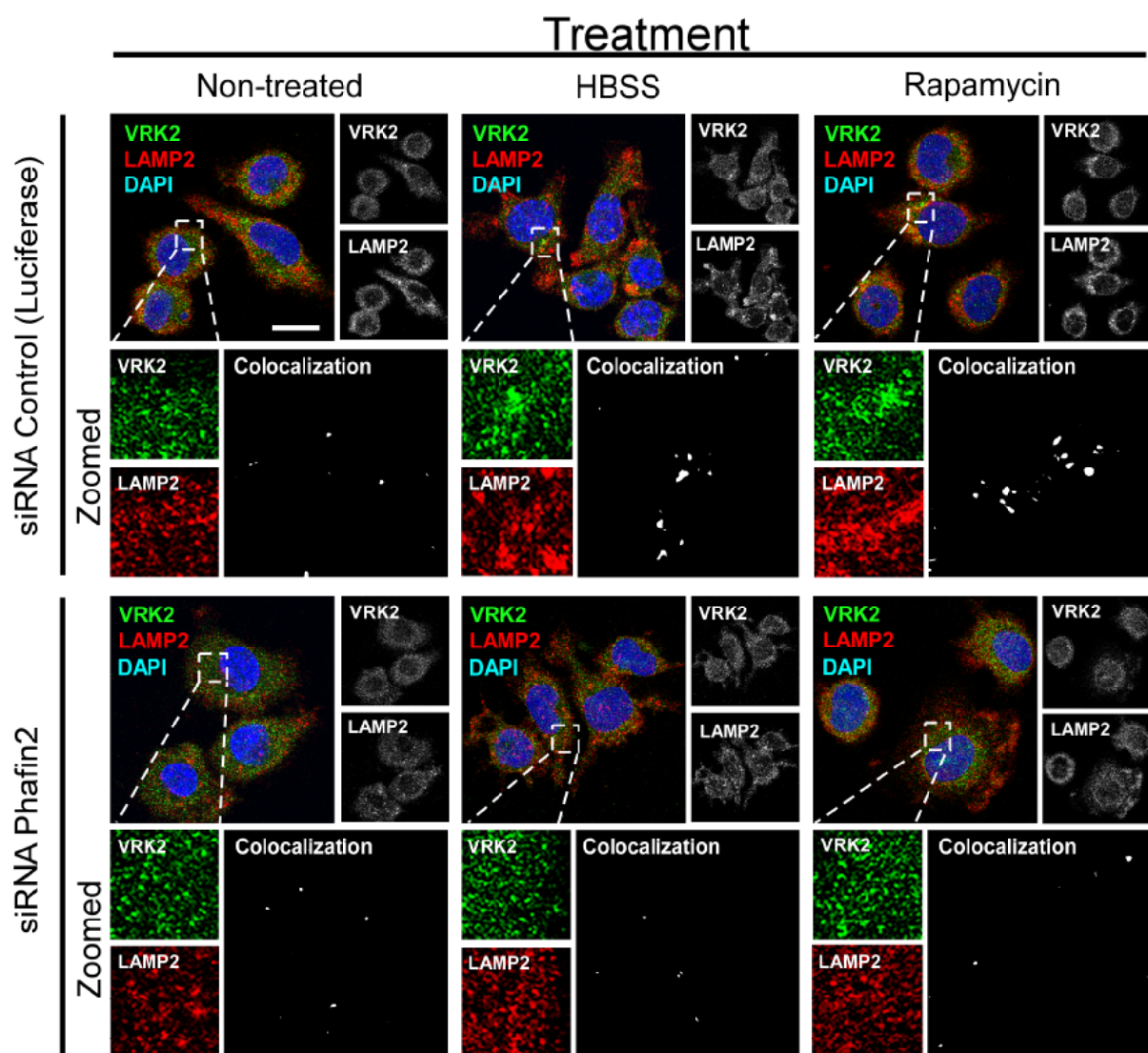

b

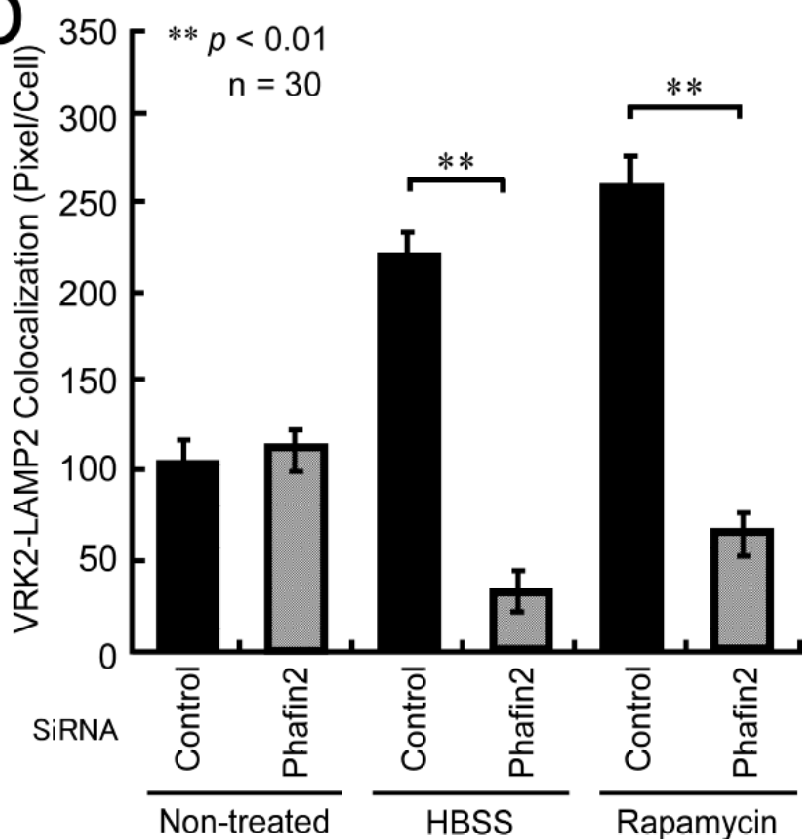

c

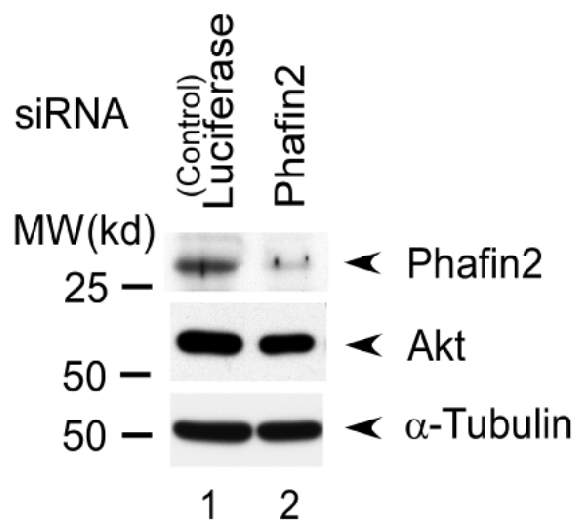

Supplement: Supplementary file 6 — Supplemental Fig 5 [file 41388_2018_330_MOESM6_ESM.pdf]

# Supplemental data 6

Hirata et al.,

Non-treated

HBSS

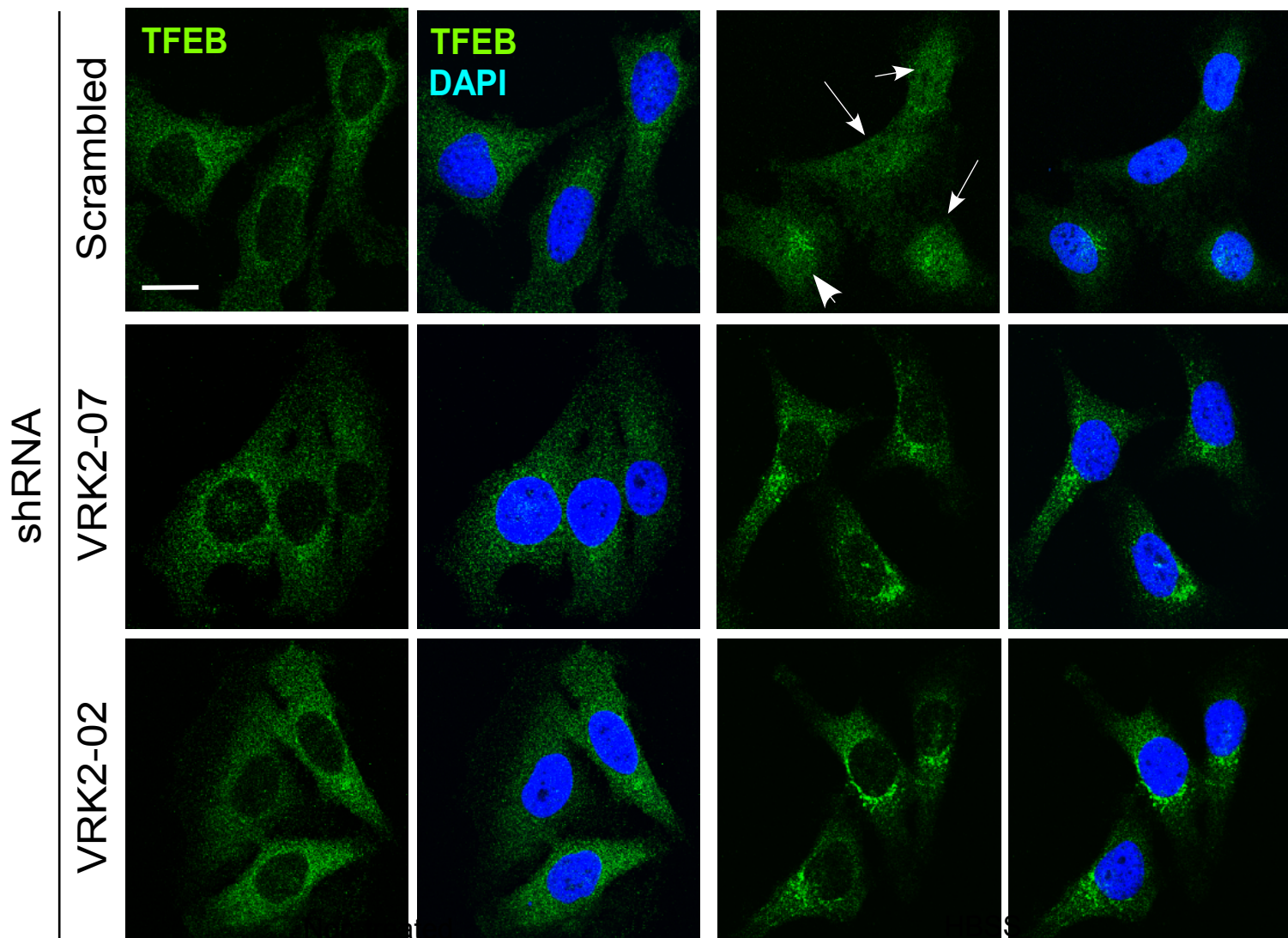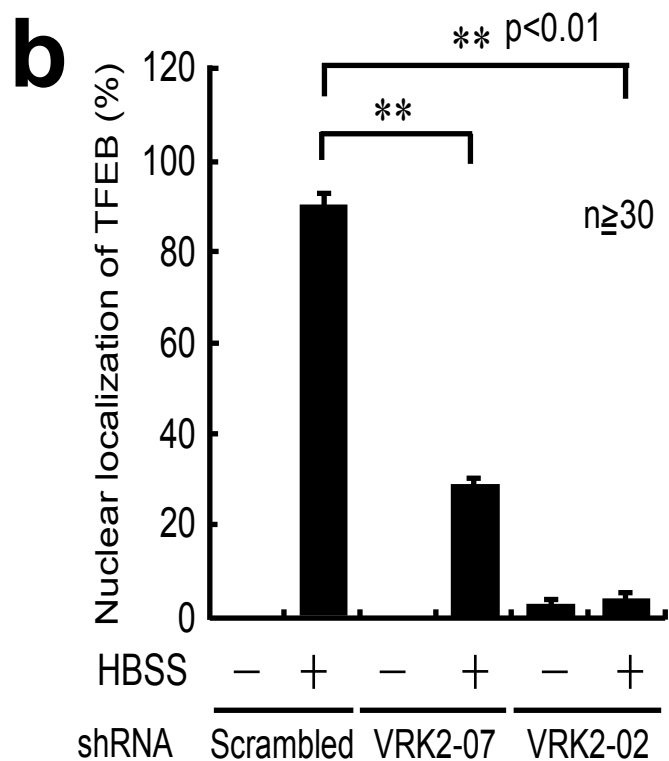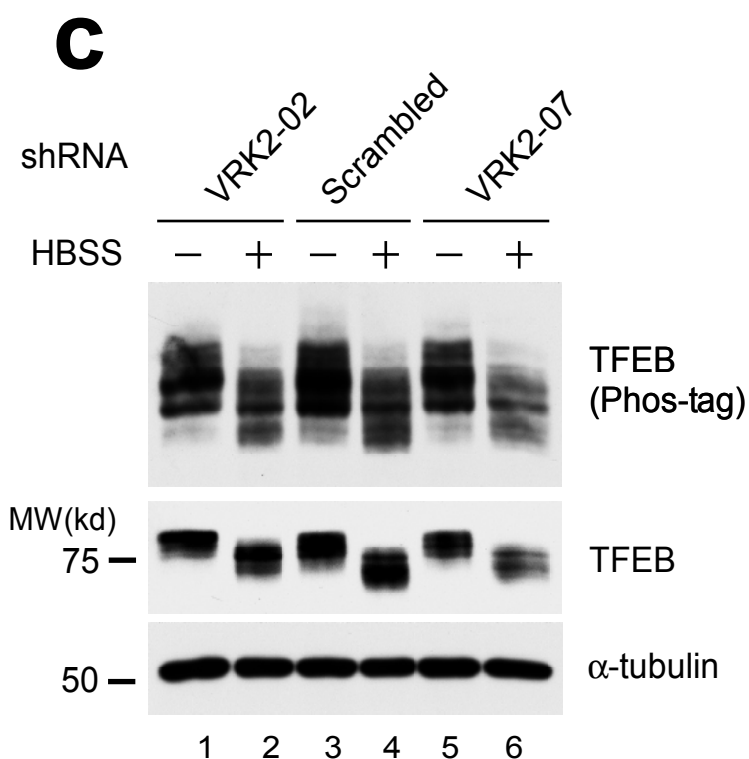

Supplement: Supplementary file 7 — Supplemental Fig 6 [file 41388_2018_330_MOESM7_ESM.pdf]

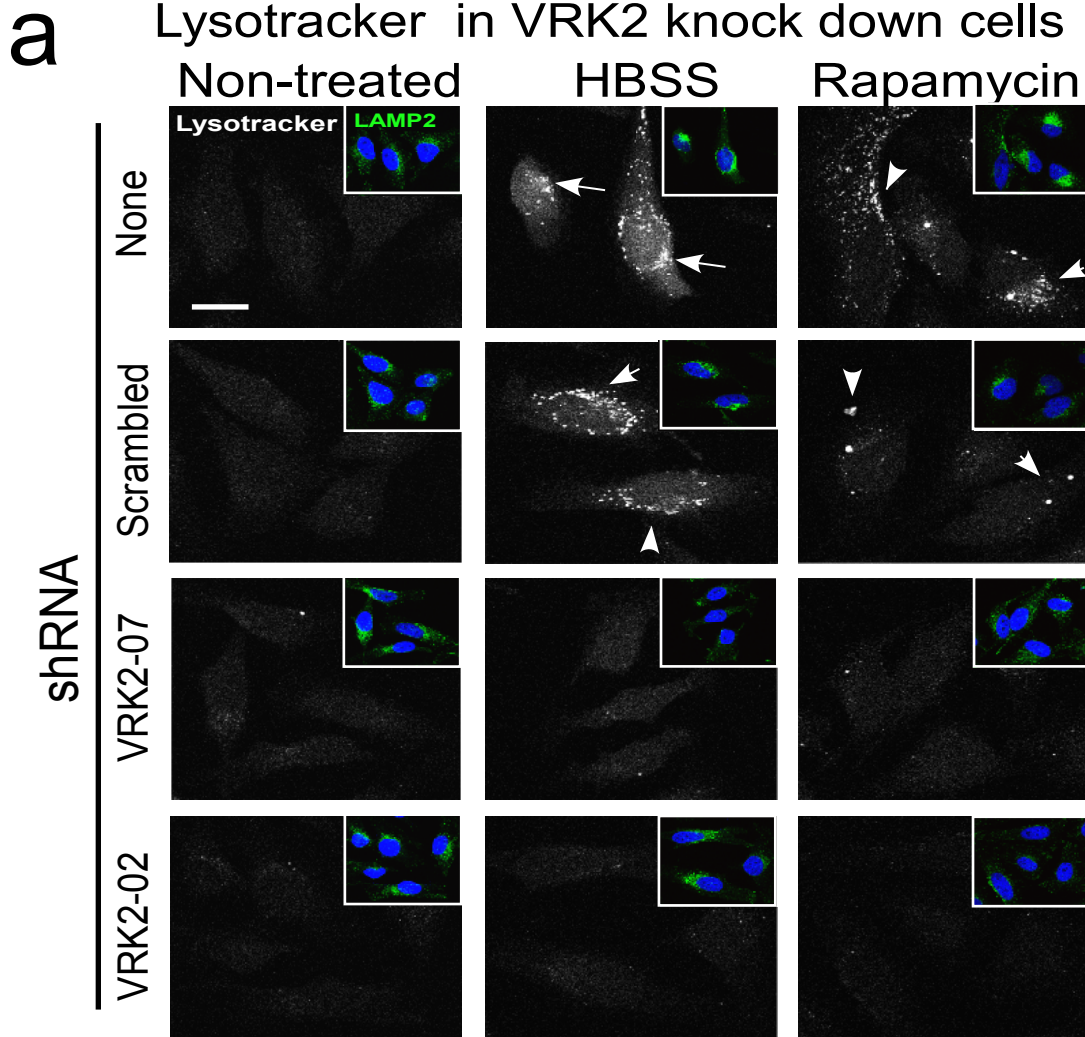

**b** Growth curve of VRK2A-overexpression

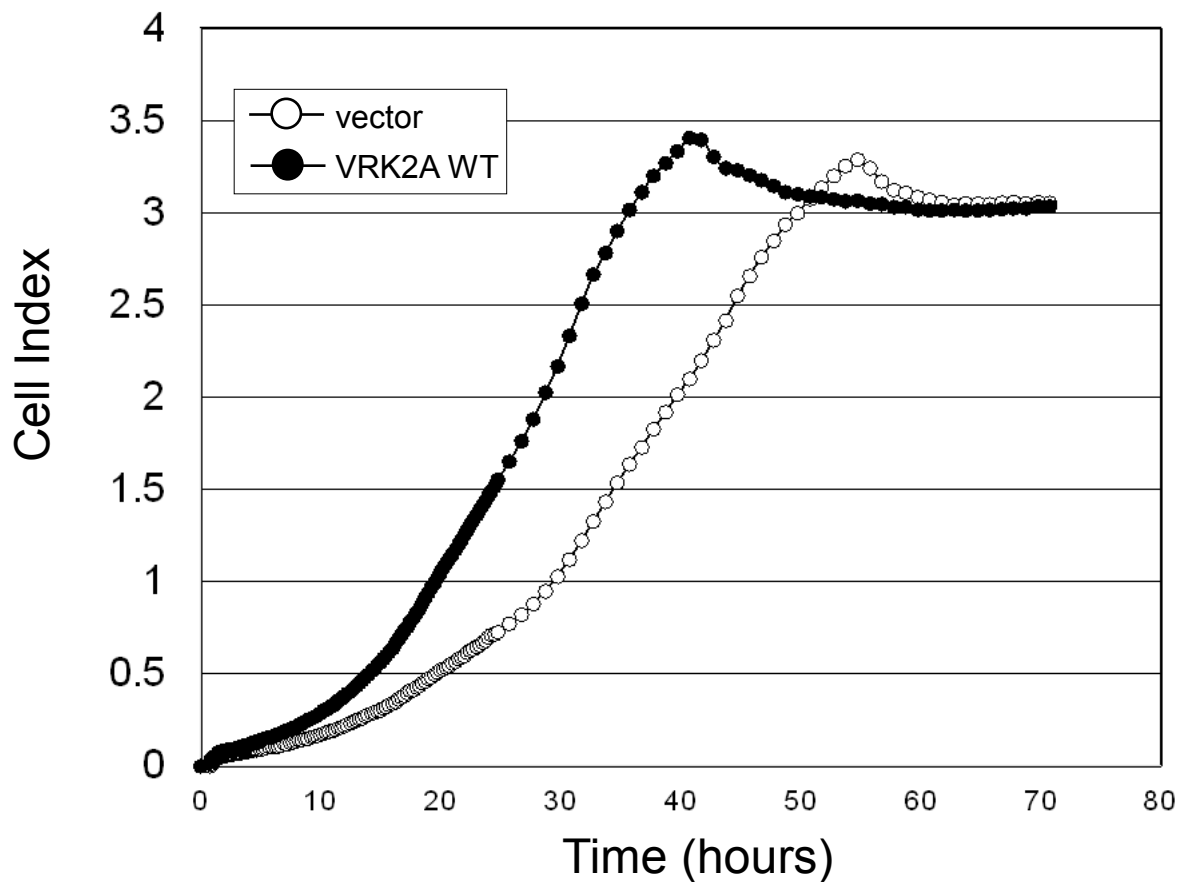

Supplement: Supplementary file 8 — Supplemtal Fig 7 [file 41388_2018_330_MOESM8_ESM.pdf]
