# Supplementary material for: Functional characterization of lysosomal interaction of Akt with VRK2
Source: Oncogene. 2018 Jun 5;37(40):5367–86. doi: 10.1038/s41388-018-0330-0 (PMC6172193; doi:10.1038/s41388-018-0330-0)

Supplemental data 2 (Fig. 1 & 2 related)

a

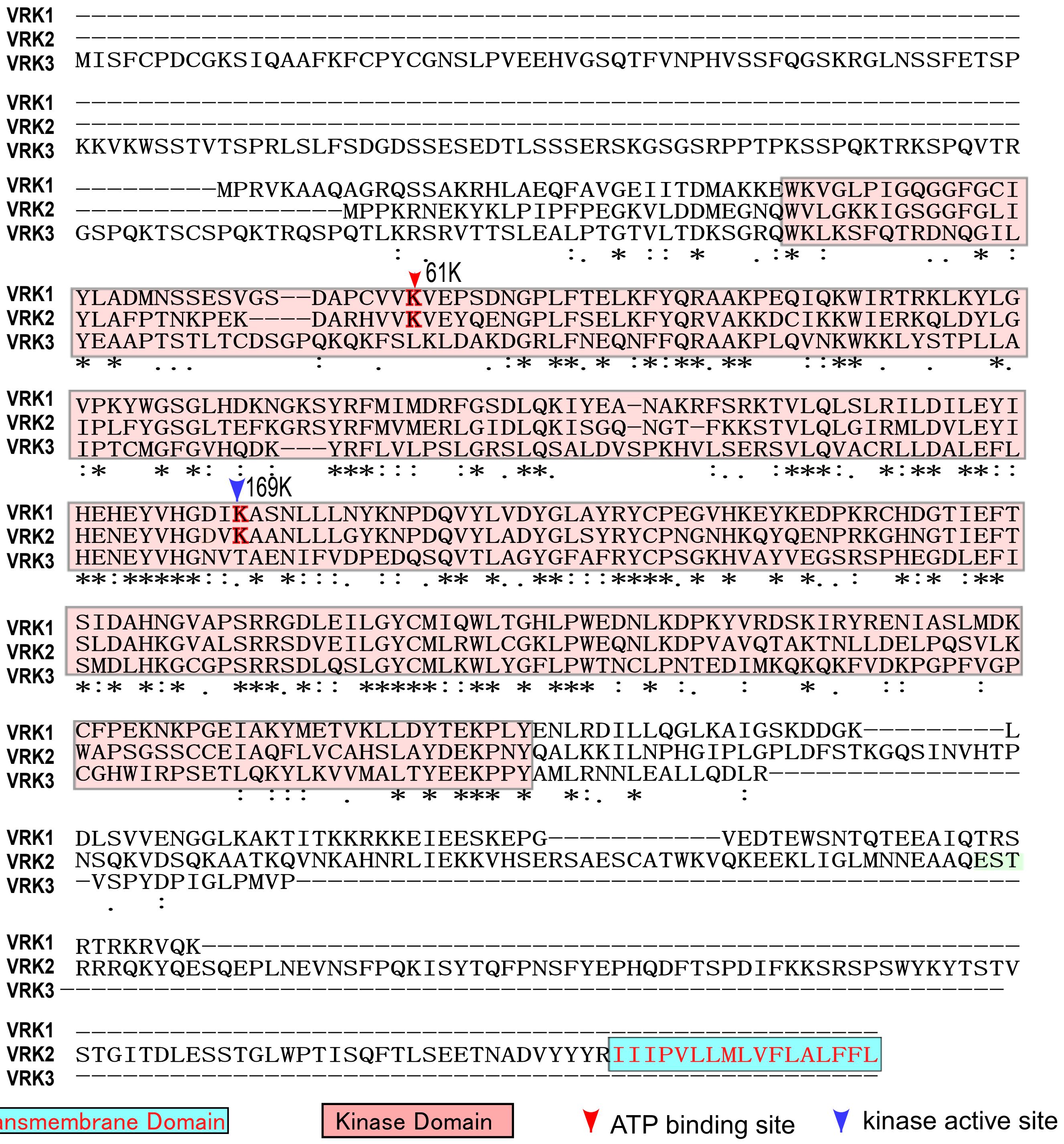

b

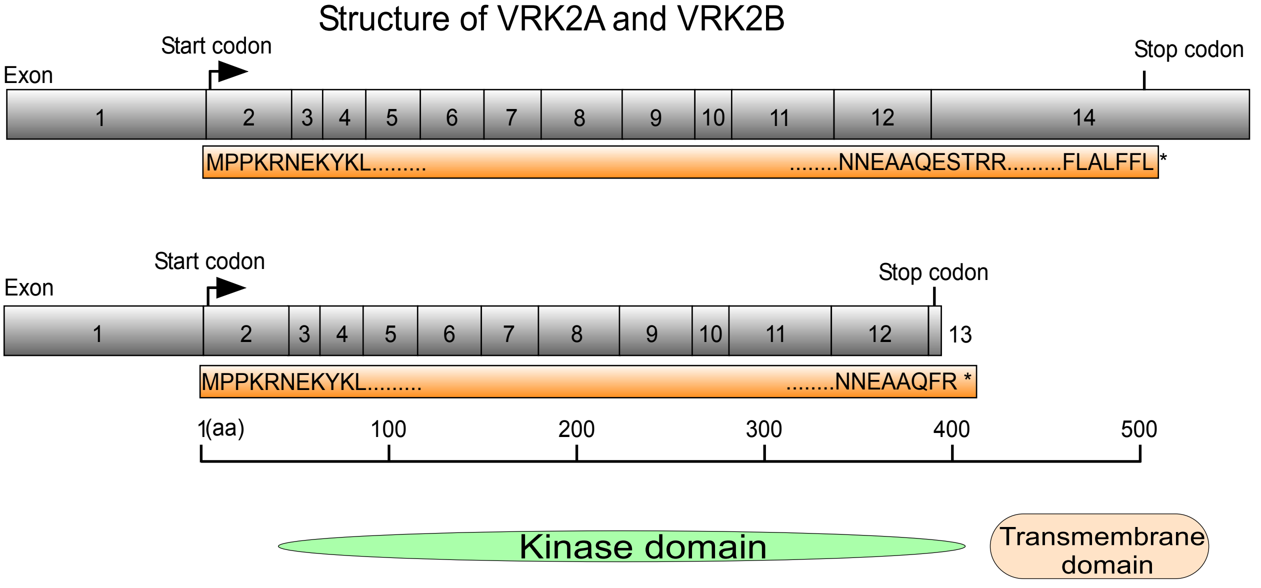

Supplement: Supplementary file 3 — Supplemental Fig 2 [file 41388_2018_330_MOESM3_ESM.pdf]
